# Ethylene precisely regulates anthocyanin synthesis in apple via a module comprising MdEIL1, MdMYB1, and MdMYB17

**DOI:** 10.1093/hr/uhac034

**Published:** 2022-02-19

**Authors:** Shuo Wang, Li-Xian Li, Zhen Zhang, Yue Fang, Dan Li, Xue-Sen Chen, Shou-Qian Feng

**Affiliations:** 1 College of Horticulture Science and Engineering, Shandong Agricultural University, Tai’an, Shandong 271018, China; 2 State Key Laboratory of Crop Biology, Tai’an, Shandong 271018, China

## Abstract

Ethylene regulates anthocyanin synthesis in ripening apple fruit via the antagonistic activities of the R2R3-MYB repressors and activators. However, the molecular mechanism underlying this process remains unknown. In this study, ethylene significantly induced the expression of the R2R3-MYB gene *MdMYB17* in apple fruit. Moreover, MdMYB17 was revealed to be an important repressor of anthocyanin synthesis. Specifically, MdMYB17 binds directly to the promoters of the ethylene-induced genes *MdMYB1* and *MdEIL1*, which encode positive regulators of anthocyanin synthesis, and represses their expression. Additionally, MdMYB1 and MdEIL1 bind to the *MdMYB17* promoter to activate its expression. Thus, MdMYB17, MdMYB1, and MdEIL1 form a regulatory module that controls the expression of the corresponding genes. MdMYB17 interacts with MdEIL1. The interaction between MdMYB17 and MdEIL1 attenuates the regulatory effects of MdMYB17 on *MdMYB1* and *MdEIL1* as well as the regulatory effects of MdEIL1 on *MdMYB17*. Overall, our results reveal the molecular mechanisms by which MdMYB17, MdMYB1, and MdEIL1 finely mediate ethylene-regulated anthocyanin synthesis in apple fruit.

## Introduction

Anthocyanins, which are flavonoids that are widely distributed in plants, have important biological functions. For example, they are responsible for the coloration of tissues and organs, while also helping to mediate pollination and reproduction and enhancing plant stress resistance [1–4]. The biosynthesis of anthocyanins begins in the phenylalanine pathway and is catalyzed by a series of enzymes encoded by structural genes, including phenylalanine ammonia lyase (PAL), chalcone synthase (CHS), chalcone isomerase (CHI), flavanone 3-hydroxylase (F3H), dihydroflavonol 4-reductase (DFR), anthocyanin synthase, and UDP-glucose/flavonoid 3-*O*-glucosyltransferase (UFGT) [5]. The expression of these structural genes is regulated by the MYB–bHLH–WDR protein complex, which is composed of an R2R3-MYB transcription factor (TF), a basic helix–loop–helix (bHLH) TF, and a WD repeat protein [6, 7]. The R2R3-MYB TFs are key regulators of anthocyanin synthesis and the spatiotemporal expression of the related structural genes. The anthocyanin-related R2R3-MYB TFs have been extensively studied in plants [8].

The R2R3-MYB TFs form one of the largest TF families in plants. The 126 R2R3-MYB TFs identified in *Arabidopsis* have been divided into 22 subgroups on the basis of their structures [9–11]. The R2R3-MYB TFs in subgroups 4 and 6 play important roles in the regulation of anthocyanin synthesis. More specifically, the R2R3-MYB TFs in subgroup 6 positively regulate anthocyanin synthesis, whereas the R2R3-MYB TFs in subgroup 4 negatively regulate anthocyanin synthesis [12–14]. Many homologs of the genes encoding the R2R3-MYB TFs in subgroups 4 and 6 have been identified in other plants; the functions of these genes are highly conserved among plant species. For example, in apple, *MdMYB1* (subgroup 6) encodes a key activator of anthocyanin synthesis [15], whereas *MdMYB16* (subgroup 4) encodes a repressor of anthocyanin synthesis [16, 17].

Anthocyanin synthesis is influenced by multiple developmental and environmental factors. Recent studies revealed that R2R3-MYB TFs regulate anthocyanin synthesis by responding to endogenous and exogenous signals. High temperatures and nitrate contents inhibit anthocyanin synthesis by decreasing the expression of R2R3-MYB activators of anthocyanin synthesis [13, 16, 18]. In contrast, exposures to light, low temperatures, ethylene, and jasmonic acid promote anthocyanin synthesis by inducing the expression of R2R3-MYB activators [19–23]. In a recent investigation, Ni *et al*. [24] showed that ethylene inhibits anthocyanidin synthesis in pear fruit by down-regulating the expression of the R2R3-MYB activators *PpMYB10* and *PpMYB114* and up-regulating the expression of the R2R3-MYB repressor *PpMYB140*. Therefore, the regulatory effects of ethylene on anthocyanin synthesis vary significantly among species and will need to be further explored.

Ethylene is an important hormone that regulates fruit ripening. In climacteric fruits, a sudden increase in respiration intensity accompanied by a peak in ethylene release during the ripening period rapidly accelerates fruit ripening [25]. Dramatic changes in fruit characteristics occur, including color changes (anthocyanin accumulation), starch degradation, and the accumulation of soluble sugars and aromatic compounds [26]. Ethylene biosynthesis and the associated signaling pathways have been thoroughly studied in the model plants tomato and *Arabidopsis*. Ethylene synthesis is catalyzed by 1-aminocyclopropane-1-carboxylic acid synthase (ACS) and 1-aminocyclopropane-1-carboxylic acid oxidase (ACO) [27]. In signal transduction pathway, ethylene was sensed by its receptors, including ethylene receptor 1 (ETR1), ETR2, ethylene response sensor 1 (ERS1), ERS2, and ethylene insensitive 4 (EIN4), and then the signal was delivered to the last component ethylene responsive factor (ERF) TFs by EIN2 and EIN3, thereby activating the ethylene response to modulate various growth and developmental processes [27, 28].

During fruit ripening, there is a close link between ethylene release and anthocyanin accumulation. In apple, MdEIL1, which is a critical component of the ethylene signaling pathway, binds directly to the *MdMYB1* promoter to induce expression, which in turn promotes anthocyanin accumulation and fruit coloration. Additionally, MdMYB1 induces ethylene production by activating MdERF3 to up-regulate *MdACS* expression [22]. MdMYB10 (an allelomorph of MdMYB1) can activate the expression of *MdERF106* [29]. MdEIL1, MdMYB1, and MdERF3 form a positive regulatory loop that mediates ethylene biosynthesis and anthocyanin accumulation [22]. However, in pear, ethylene inhibits anthocyanin synthesis by activating the expression of a negative regulator of anthocyanin synthesis (*PpMYB140*) through PpERF105 [24]. The R2R3-MYB repressor gene *PpMYB18* is highly expressed in ripe peach fruit with high anthocyanin concentrations [30]. Therefore, ethylene regulates anthocyanin synthesis via the antagonistic activities of the R2R3-MYB repressors and activators. However, the underlying mechanism remains unclear.

Apple is a typical respiratory climacteric fruit, in which respiration and ethylene production markedly increase during the ripening stage [31]. Many genes associated with ethylene biosynthesis and the related signaling pathways were recently identified in apple, including *MdACS1*, *MdACO1*, *MdERF2*, *MdERF3*, and *MdEIL1* [32, 33]. Previous studies showed that apple fruit ripening is accompanied by an increase in ethylene release. Moreover, substantial amounts of anthocyanins begin to accumulate, which leads to fruit coloration. During the fruit ripening stage, ethylene induces anthocyanin synthesis through the MdEIL1–MdMYB1 signaling pathway [22]. However, the molecular mechanism of ethylene regulating anthocyanin synthesis by R2R3-MYB repressors in apple fruit remains unclear. In this study, our analyses indicated that MdMYB17 plays an important regulatory role in ethylene-mediated anthocyanin synthesis in apple. More specifically, MdMYB17 together with MdMYB1 and MdEIL1 forms a complex feedback regulatory network that finely controls anthocyanin synthesis. Moreover, MdMYB17 interacts with MdEIL1 to attenuate the regulatory effects of MdMYB17 on *MdMYB1* and *MdEIL1* as well as the regulatory effects of MdEIL1 on *MdMYB1* and *MdMYB17*. Hence, MdMYB17 negatively regulates anthocyanin synthesis and interacts with MdMYB1 and MdEIL1 to precisely control ethylene-regulated anthocyanin synthesis and maintain appropriate anthocyanin levels. The results of this study provide new insights into the molecular basis of ethylene-regulated anthocyanin synthesis in apple fruit.

**Figure 1:**
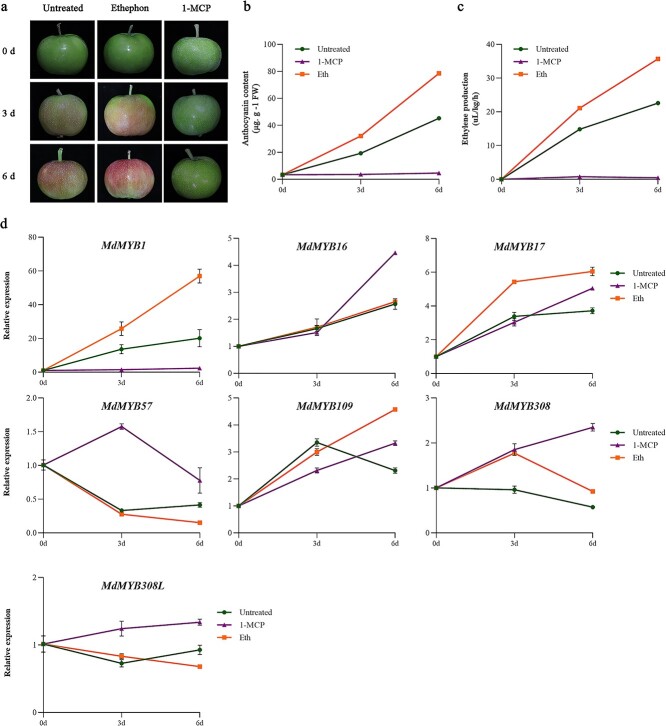
Effects of ethephon and 1-MCP on anthocyanin synthesis in ‘Geneva Early’ apple fruit. **a** Phenotypes of ‘Geneva Early’ fruit treated with ethephon (Eth) and 1-MCP. **b** Anthocyanin contents of ‘Geneva Early’ fruit treated with ethephon and 1-MCP. **c** Ethylene production of ‘Geneva Early’ fruit treated with ethephon and 1-MCP. **d** Expression levels of *MdMYB1* and R2R3-MYB TF genes of subfamily 4 in ‘Geneva Early’ apple fruit treated with ethephon and 1-MCP. Samples were analyzed at 0, 3, and 6 days after the ethephon and 1-MCP treatments. Data are presented as the mean ± standard deviation of three independent biological replicates.

## Results

### Ethylene promotes *MdMYB1* and *MdMYB17* expression and anthocyanin synthesis in apple fruit

To verify the effect of ethylene on the expression of anthocyanin synthesis-related R2R3-MYB activators and repressors in apple, we collected ‘Geneva Early’ apple fruits at 60 days after full bloom (DAFB) and then treated them with ethephon (1000 mg l^−1^) or the ethylene inhibitor 1-methylcyclopropene (1-MCP; 1 μl l^−1^). The treated fruits were stored for 6 days in an incubator set at 24°C with a light intensity of 20 000 lux. During storage, we analyzed ethylene release, anthocyanin accumulation in the pericarp, and the expression levels of *MdMYB1*. Consistent with the findings of an earlier study [22], the ethephon treatment significantly induced ethylene release, anthocyanin synthesis, and *MdMYB1* expression in the apple fruit, whereas the 1-MCP treatment had the opposite effects (Fig. 1a–d). These results suggest that MdMYB1 contributes to ethylene-regulated anthocyanin synthesis in apple fruit. We used *Arabidopsis* R2R3-MYB subfamily 4 genes to search the GDR (https://www.rosaceae.org/) databases, and obtained six R2R3-MYB subfamily 4 genes in apple genome (GenBank accession numbers are listed in Supplementary Data Table S2). Regarding the subfamily 4 R2R3-MYB TF genes in apple, ethephon and 1-MCP induced the expression of *MdMYB17*, *MdMYB16*, *MdMYB308*, and *MdMYB109*, but not *MdMYB57* and *MdMYB308L*. Among the above genes, the *MdMYB17* expression level was up-regulated the most, implying that MdMYB17 may have an important regulatory role during ethylene-induced anthocyanin synthesis (Fig. 1d). Consequently, we selected *MdMYB17* for the subsequent functional investigation.

### *MdMYB17* inhibits anthocyanin synthesis and fruit coloration in apple

**Figure 2:**
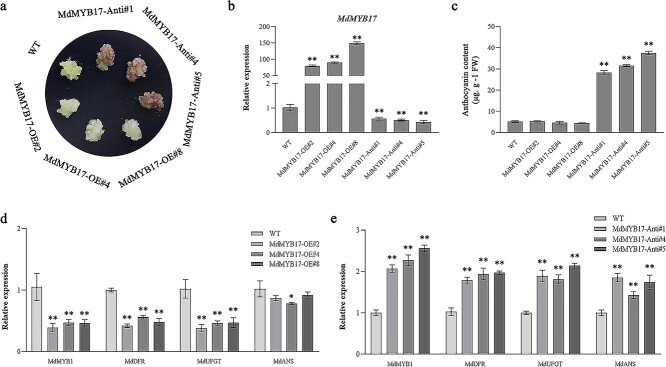
MdMYB17 inhibits anthocyanin synthesis in ‘Orin’ apple calli. **a** Phenotypes of ‘Orin’ apple calli incubated at 14°C and light (20 000 lux) for 10 days. *MdMYB17*-Anti, *MdMYB17*-antisense suppression. **b** Expression levels of *MdMYB17* in ‘Orin’ apple calli. **c** Anthocyanin contents of ‘Orin’ apple calli. FW, fresh weight. **d, e** Expression levels of anthocyanin-related genes in ‘Orin’ apple calli. Data are presented as the mean ± standard deviation of three independent biological replicates. Asterisks indicate significant differences, as determined by Student’s *t*-test (^*^P < .05, ^**^P < .01).

To further clarify the MdMYB17 function related to apple anthocyanin synthesis, we generated apple calli overexpressing *MdMYB17* (*MdMYB17*-OE) and calli in which *MdMYB17* expression was suppressed (*MdMYB17*-RNAi). The *MdMYB17*-RNAi apple calli were red, but the *MdMYB17*-OE and wild-type (WT) calli were not (Fig. 2a–c). Compared with the corresponding expression levels in the WT calli, *MdMYB1*, *MdDFR*, and *MdUFGT* were expressed at lower and higher levels in the *MdMYB17*-OE and *MdMYB17*-RNAi calli, respectively (Fig. 2d and e).

We also analyzed transient gene expression in apple fruit. Compared with control fruit, overexpression of *MdMYB17* inhibited anthocyanin accumulation at the apple fruit injection site. Additionally, the *MdMYB1*, *MdDFR*, and *MdUFGT* expression levels were significantly down-regulated at the *MdMYB17*-pRI injection site (Supplementary Data Fig. S1). In contrast, silencing of *MdMYB17* promoted anthocyanin accumulation at the apple fruit injection site. Moreover, the *MdMYB1*, *MdDFR*, *MdUFGT*, and *MdANS* expression levels were significantly up-regulated at the *MdMYB17*-TRV injection site (Supplementary Data Fig. S2). These results suggest that MdMYB17 may negatively regulate anthocyanin synthesis in apple.

### MdMYB17 represses the transcription of *MdMYB1*, *MdDFR*, and *MdUFGT* by binding to their promoters

**Figure 3:**
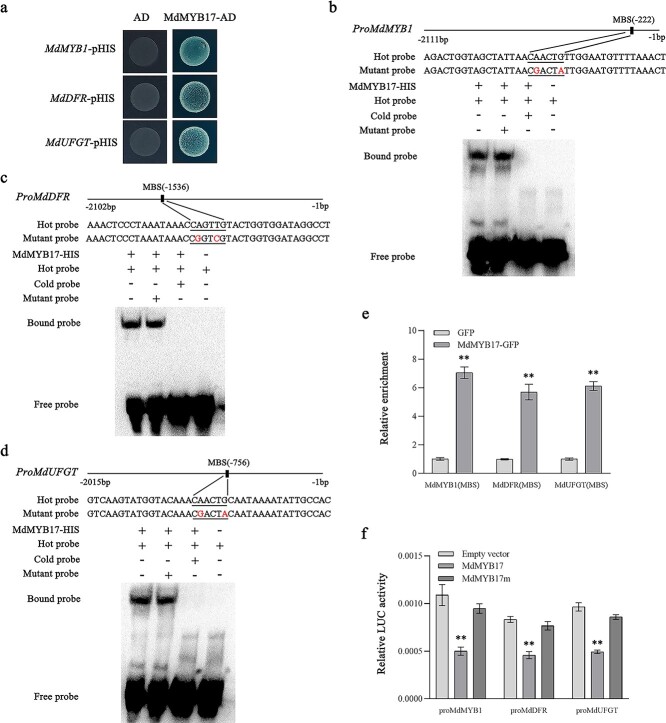
MdMYB17 inhibits *MdMYB1*, *MdDFR*, and *MdUFGT* transcription. **a** Y1H analysis indicating that MdMYB17 binds to the *MdMYB1*, *MdDFR*, and *MdUFGT* promoters. The 3-AT concentration was 130 ng ml^−1^. The empty vector and the *MdMYB1*, *MdDFR*, and *MdUFGT* promoters were used as negative controls. **b**–**d** EMSA analysis indicating that MdMYB17 binds to the MBS motifs in the *MdMYB1* (**b**), *MdDFR* (**c**), and *MdUFGT* (**d**) promoters. The hot probe was a biotin-labeled promoter fragment containing the MBS motif, whereas the cold probe was an unlabeled competitive probe (300-fold probe concentration). Mutant probes were unlabeled hot probes containing two nucleotide mutations. **e** ChIP–qPCR analysis indicating the *in vivo* interaction between MdMYB17 and the *MdMYB1*, *MdDFR*, and *MdUFGT* promoters. ‘Orin’ apple callus overexpressing the GFP sequence was used as a negative control. The ChIP assay was completed using three replicates. **f** LUC activity analysis indicating that MdMYB17 inhibits *MdMYB1*, *MdDFR*, and *MdUFGT* promoter activities. MdMYB17m, MdMYB17 protein sequence lacking the C2/EAR motif. Data are presented as the mean ± standard deviation of three independent biological replicates. Asterisks indicate significant differences, as determined by Student’s *t*-test (^**^P < .01).

To investigate the regulatory relationships between MdMYB17 and *MdMYB1*, *MdDFR*, and *MdUFGT*, we analyzed the binding of MdMYB17 to the *MdMYB1*, *MdDFR*, and *MdUFGT* promoters in yeast one-hybrid (Y1H) assays. The Y1H results indicated that MdMYB17 binds to the promoters of all three genes (Fig. 3a). The interactions were verified in an electrophoretic mobility shift assay (EMSA). The *MdMYB1*, *MdDFR*, and *MdUFGT* promoters contain one MYB-binding site (MBS) motif. In this study, we used biotinylated MBS-containing promoter fragments as probes. The EMSA confirmed that MdMYB17 can bind to the *MdMYB1* (Fig. 3b, lane 1), *MdDFR* (Fig. 3c, lane 1), and *MdUFGT* (Fig. 3d, lane 1) promoters. This binding was unaffected by supplementation with a competitor probe containing mutations. Accordingly, MdMYB17 binds to the MBS motif in the *MdMYB1* (Fig. 3b, lane 2), *MdDFR* (Fig. 3c, lane 2), and *MdUFGT* (Fig. 3d, lane 2) promoters.

To assess the *in vivo* binding of MdMYB17 to the *MdMYB1*, *MdDFR*, and *MdUFGT* promoters, a chromatin immunoprecipitation and quantitative real-time PCR (ChIP–qPCR) analysis was performed using 35S::MdMYB17-GFP transgenic apple calli and empty green fluorescent protein (GFP) vector transgenic apple calli as controls. The results reflected the *in vivo* binding of MdMYB17 to the promoters of all three genes (Fig. 3e).

We next analyzed the effect of MdMYB17 on the *MdMYB1*, *MdDFR*, and *MdUFGT* promoter activities by conducting luciferase (LUC) assays. The *MdMYB1*, *MdDFR*, and *MdUFGT* promoter sequences were inserted into the pFRK1-LUC vector so they were fused to the LUC reporter gene. The *MdMYB17* coding sequence (CDS) was inserted into the pHBT-AvrRpm1 vector as an effector. Compared with the controls, the co-expression of 35S::MdMYB17 with proMdMYB1::LUC, proMdMYB1::LUC, and proMdMYB1::LUC resulted in significantly weaker LUC activity (Fig. 3f). These results imply that MdMYB17 represses *MdMYB1*, *MdDFR*, and *MdUFGT* transcription.

Many R2R3-MYB repressors contain the conserved C2/EAR repressor motif in their C terminus [14], and a conserved LxLxL C2/EAR was also found in the C terminus of MdMYB17 (Supplementary Data Fig. S3). We conducted LUC assays to investigate the effect of the C2/EAR motif on the *MdMYB1*, *MdDFR*, and *MdUFGT* promoter activities. The mutated *MdMYB17* sequence lacking the C2/EAR motif (MdMYB17m) was inserted into the pHBT-AvrRpm1 vector as an effector. Compared with MdMYB17, the inhibitory effect of MdMYB17m on *MdMYB1*, *MdDFR*, and *MdUFGT* transcription was significantly reduced (Fig. 3f). These findings suggest that the C2/EAR motif in MdMYB17 has a repressing effect.

### MdMYB1 positively regulates the expression of *MdMYB17*

**Figure 4:**
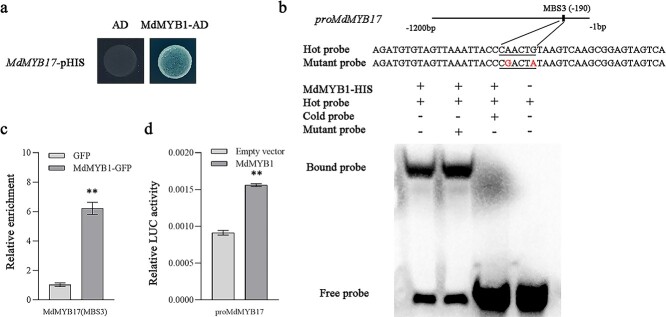
MdMYB1 promotes *MdMYB17* transcription. **a** Y1H analysis indicating that MdMYB1 binds to the *MdMYB17* promoter. The concentration of 3-AT was 150 ng ml^−1^. The empty vector and the *MdMYB17* promoter were used as negative controls. **b** EMSA analysis indicating that MdMYB1 binds to the MBS3 motif in the *MdMYB17* promoter. The hot probe was a biotin-labeled promoter fragment containing the MBS motif, whereas the cold probe was an unlabeled competitive probe (200-fold probe concentration). The mutant probe was unlabeled hot probe containing two nucleotide mutations. **c** ChIP–qPCR analysis indicating the *in vivo* interaction between MdMYB1 and the *MdMYB17* promoter. ‘Orin’ apple callus overexpressing the GFP sequence was used as a negative control. The ChIP assay was completed using three replicates. **d** LUC activity analysis indicating that MdMYB1 activates the *MdMYB17* promoter. Data are presented as the mean ± standard deviation of three independent biological replicates. Asterisks indicate significant differences, as determined by Student’s *t*-test (^**^P < .01).

As mentioned above, ethephon can induce the expression of both *MdMYB1* and *MdMYB17*, and the *MdMYB1* and *MdMYB17* expression levels are significantly correlated (*R*^2^ = 0.7877) (Supplementary Data Fig. S4). Therefore, we speculated that MdMYB1 may activate the transcription of *MdMYB17*. To investigate the regulatory relationship between MdMYB1 and *MdMYB17*, we generated apple calli overexpressing *MdMYB1* (*MdMYB1*-OE). Compared with the WT calli, the overexpression of *MdMYB1* significantly increased anthocyanin accumulation and *MdMYB17* expression in the transgenic calli (Supplementary Data Fig. S5). To verify the regulatory relationship between MdMYB1 and *MdMYB17*, we performed a Y1H assay, which confirmed that MdMYB1 binds to the *MdMYB17* promoter (Fig. 4a), which includes three MBS motifs (MBS1, MBS2, and MBS3). The EMSA results indicated that MdMYB1 binds to MBS3 in the *MdMYB17* promoter (Fig. 4b; Supplementary Data Fig. S6). The ChIP–qPCR analysis revealed the *in vivo* binding of MdMYB1 to the MBS3 motif of the *MdMYB17* promoter (Fig. 4c). The subsequent analysis of the regulatory effect of MdMYB1 on the *MdMYB17* promoter in an LUC assay indicated that MdMYB1 promotes *MdMYB17* expression (Fig. 4d).

### MdEIL1 positively regulates the expression of *MdMYB17*, whereas MdMYB17 represses the expression of *MdEIL1*

**Figure 5:**
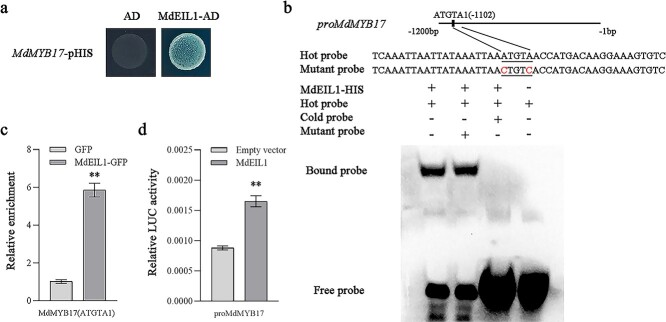
MdEIL1 promotes *MdMYB17* transcription. **a** Y1H analysis indicating that MdEIL1 binds to the *MdMYB17* promoter. The concentration of 3-AT was 120 ng ml^−1^. The empty vector and the *MdMYB17* promoter were used as negative controls. **b** EMSA analysis indicating that MdEIL1 binds to the ATGTA1 motif in the *MdMYB17* promoter. The hot probe was a biotin-labeled promoter fragment containing the ATGTA motif, whereas the cold probe was an unlabeled competitive probe (300-fold probe concentration). The mutant probe was unlabeled hot probe containing two nucleotide mutations. **c** ChIP–qPCR analysis indicating the *in vivo* interaction between MdEIL1 and the *MdMYB17* promoter. ‘Orin’ apple callus overexpressing the GFP sequence was used as a negative control. The ChIP assay was completed using three replicates. **d** LUC activity analysis indicating that MdEIL1 activates the *MdMYB17* promoter. Data are presented as the mean ± standard deviation of three independent biological replicates. Asterisks indicate significant differences, as determined by Student’s *t*-test (^**^P < .01).

Earlier studies identified *MdEIL1* as a core gene for ethylene-regulated anthocyanin synthesis during apple fruit ripening [22]. In this study, we generated ‘Orin’ apple calli overexpressing *MdEIL1*. Consistent with previous results, *MdEIL1* overexpression significantly increased anthocyanin accumulation and up-regulated *MdMYB17* expression in transgenic apple calli (Supplementary Data Fig. S7). Furthermore, *MdEIL1* expression was substantially up-regulated by the ethephon treatment (Supplementary Data Fig. S8). A Y1H assay performed to further clarify the regulatory relationship between MdEIL1 and *MdMYB17* demonstrated that MdEIL1 binds to the *MdMYB17* promoter (Fig. 5a), which contains two ATGTA motifs (ATGTA1 and ATGTA2). An EMSA indicated that MdEIL1 binds to the ATGTA1 motif of the *MdMYB17* promoter (Fig. 5b; Supplementary Data Fig. S9). The ChIP–qPCR analysis confirmed the *in vivo* binding of MdEIL1 to the ATGTA1 motif of the *MdMYB17* promoter (Fig. 5c). The LUC assay results suggested that MdEIL1 promotes *MdMYB17* expression (Fig. 5d).

**Figure 6:**
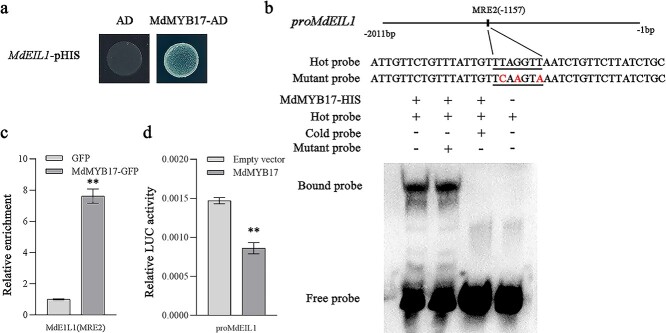
MdMYB17 inhibits *MdEIL1* transcription. **a** Y1H analysis indicating that MdMYB17 binds to the *MdEIL1* promoter. The concentration of 3-AT was 200 ng ml^−1^. The empty vector and the *MdEIL1* promoter were used as negative controls. **b** EMSA analysis indicating that MdMYB17 binds to the MRE2 motif in the *MdEIL1* promoter. The hot probe was a biotin-labeled promoter fragment containing the MRE motif, whereas the cold probe was an unlabeled competitive probe (300-fold probe concentration). The mutant probe was unlabeled hot probe containing three nucleotide mutations. **c** ChIP–qPCR analysis indicating the *in vivo* interaction between MdMYB17 and the *MdEIL1* promoter. ‘Orin’ apple callus overexpressing the GFP sequence was used as a negative control. The ChIP assay was completed using three replicates. **d** LUC activity analysis indicating that MdMYB17 inhibits the *MdEIL1* promoter activity. Data are presented as the mean ± standard deviation of three independent biological replicates. Asterisks indicate significant differences, as determined by Student’s *t*-test (^**^P < .01).

We also observed that *MdEIL1* was expressed at significantly lower levels in *MdMYB17*-OE apple calli than in WT calli. In contrast, *MdEIL1* was more highly expressed in *MdMYB17*-RNAi apple calli than in WT calli (Supplementary Data Fig. S10). Thus, MdMYB17 appears to repress *MdEIL1* transcription. To investigate the regulatory relationship between MdMYB17 and *MdEIL1*, we performed a Y1H assay, which revealed that MdMYB17 binds directly to the *MdEIL1* promoter (Fig. 6a). The *MdEIL1* promoter contains two MYB-recognition elements (MREs), namely MRE1 and MRE2. An EMSA indicated that MdMYB17 binds to MRE2 in the *MdEIL1* promoter (Fig. 6b; Supplementary Data Fig. S11). The ChIP–qPCR analysis confirmed the *in vivo* binding of MdMYB17 to MRE2 in the *MdEIL1* promoter (Fig. 6c). The LUC assay suggested that MdMYB17 inhibits *MdEIL1* expression (Fig. 6d).

### The interaction between MdEIL1 and MdMYB17 affects the transcription of *MdEIL1*, *MdMYB17*, and *MdMYB1*

**Figure 7:**
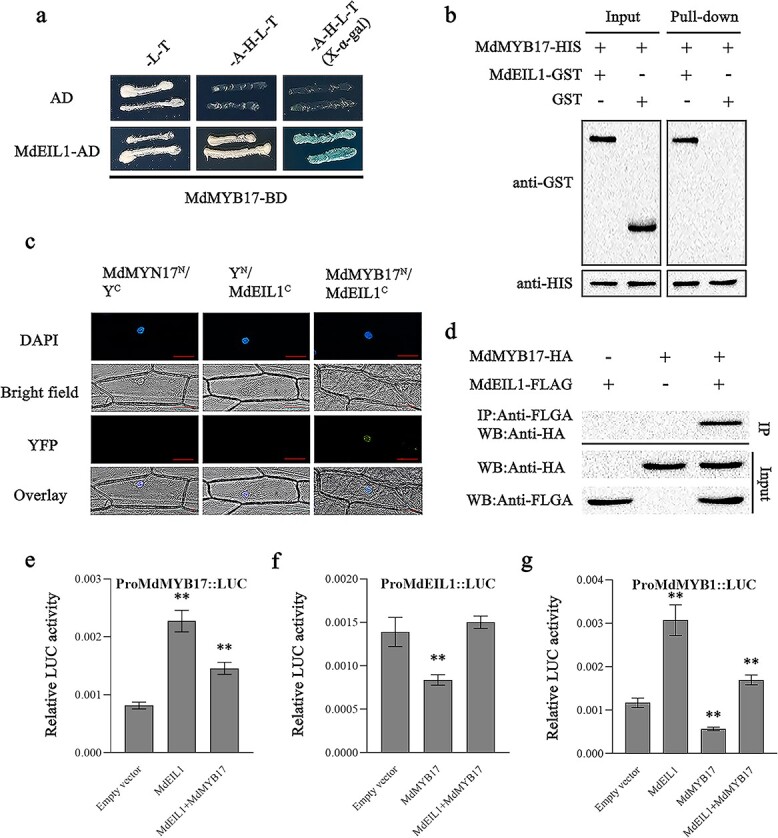
MdMYB17 interacts with MdEIL1. **a** Y2H analysis indicating that MdMYB17 interacts with MdEIL1. −L–T, SD medium lacking Leu and Trp; −A−H−L−T, SD medium lacking Leu, Trp, His, and Ade; X-α-gal, SD medium containing X-α-gal. The empty AD vectors were used as the negative control. **b** Pull-down analysis indicating that MdMYB17 interacts with MdEIL1. An anti-GST antibody was used for immunoblot analyses. **c** BiFC analysis indicating the *in vivo* interaction between MdMYB17 and MdEIL1. Empty YFPN and YFPC vectors were used as negative controls. Scale bar = 50 μm. **d** Co-IP analysis indicating the *in vivo* interaction between MdMYB17 and MdEIL1. Both MdMYB17-HA and MdEIL1-FLAG were transiently co-expressed in protoplasts prepared from ‘Orin’ apple calli. The samples were precipitated using anti-FLAG agarose beads. **e**–**g** LUC analysis indicating that MdMYB17–MdEIL1 weakens the effects of MdEIL1 or MdMYB17 on the *MdMYB17* (**e**), *MdEIL1* (**f**), and *MdMYB1* (**g**) promoters. Data are presented as the mean ± standard deviation of three independent biological replicates. Asterisks indicate significant differences, as determined by Student’s *t*-test (^**^P < .01).

As a key component of the ethylene signaling pathway, EIN3/EIL interacts with multiple functionally important proteins to regulate plant growth, development, and stress resistance [34, 35]. Therefore, MdEIL1 may interact with MdMYB17 to influence the regulatory effect of ethylene on anthocyanin synthesis. To assess this possibility, we examined the interaction between MdEIL1 and MdMYB17 in a yeast two-hybrid (Y2H) assay. The recombinant pGBKT7 plasmid containing the *MdEIL1* CDS was self-activating. Therefore, we inserted the *MdEIL1* and *MdMYB17* CDSs into the pGADT7 and pGBKT7 vectors, respectively. The Y2H assay proved that MdEIL1 can interact with MdMYB17 (Fig. 7a). We then purified the polyhistidine (His)-tagged MdMYB17 (MdMYB17-HIS) fusion protein and the glutathione-*S*-transferase (GST)-tagged MdEIL1 (MdEIL1-GST) fusion protein for pull-down assays, in which MdEIL1-GST was pulled down by MdMYB17-HIS, implying MdEIL1 interacts with MdMYB17 (Fig. 7b).

To investigate the *in vivo* interaction between MdEIL1 and MdMYB17, we performed a bimolecular fluorescence complementation (BiFC) assay. The co-expression of MdMYB17^N^ and MdEIL1^C^ in onion epidermal cells resulted in a strong yellow fluorescent protein (YFP) signal, indicative of the *in vivo* interaction between MdMYB17 and MdEIL1 (Fig. 7c). Next, we performed a co-immunoprecipitation (Co-IP) analysis using apple calli. The immunoprecipitation of MdMYB17-HA by MdEIL1-FLAG in apple calli confirmed the *in vivo* interaction between MdMYB17 and MdEIL1 (Fig. 7d).

LUC assays were completed to determine how the interaction between MdMYB17 and MdEIL1 affects *MdMYB17* or *MdEIL1* promoter activity. The LUC activity of the *MdMYB17* promoter was significantly lower when MdMYB17 and MdEIL1 were co-expressed than when only MdEIL1 was expressed (Fig. 7e). In contrast, the LUC activity of the *MdEIL1* promoter was higher when MdMYB17 and MdEIL1 were co-expressed than when only *MdMYB17* was expressed (Fig. 7f). Previous studies demonstrated that MdEIL1 directly activates *MdMYB1* expression. Therefore, we investigated how the interaction between MdMYB17 and MdEIL1 influences *MdMYB1* promoter activity. The LUC assay results indicated that the interaction between MdMYB17 and MdEIL1 attenuated the activation of the *MdMYB1* promoter by MdEIL1 and the repression of the *MdMYB1* promoter by MdMYB17 (Fig. 7g).

## Discussion

Ethylene is an important hormone that promotes fruit ripening and affects fruit quality. In recent years, a series of ethylene-related genes have been identified. The functional characterization of these genes indicated they are involved in various physiological processes, including fruit coloration [22, 36]. An ethephon treatment of apple fruit significantly enhances anthocyanin synthesis and coloration [22]. However, the molecular mechanisms by which ethylene regulates anthocyanin synthesis in ripening apple fruit have not been thoroughly characterized. In the present study, ethylene induced the expression of *MdMYB17*, which encodes an R2R3-MYB TF that represses anthocyanin synthesis. Additionally, MdMYB17 together with MdMYB1 and MdEIL1 formed a regulatory module that finely mediated the regulation of anthocyanin synthesis by ethylene in apple fruit.

### *MdMYB17* is a subfamily 4 R2R3-MYB anthocyanin synthesis repressor gene

Plants contain many R2R3-MYB TFs, which vary considerably in terms of structures and functions. R2R3-MYB TFs include crucial regulators of anthocyanin synthesis [9]. Additionally, the subgroup 6 members AtPAP1 and AtPAP2 as well as their homologs are positive regulators of anthocyanin synthesis; the underlying regulatory mechanisms have been extensively studied [10, 12]. However, the mechanisms mediating the effects of subgroup 4 R2R3-MYB TFs, which negatively regulate anthocyanin synthesis, have only recently been elucidated [14]. In the present study, we identified six genes encoding subgroup 4 R2R3-MYB TFs in the apple genome. Of these genes, *MdMYB17* was the most responsive to ethylene. Moreover, the transgenic assay confirmed that MdMYB17 can inhibit anthocyanin synthesis. Thus, MdMYB17 is important for ethylene-regulated apple fruit coloration.

The C2 repressor motif, which is also known as the EAR repression domain, contains a conserved DLNxxP or LxLxL core sequence and is present in various TFs with repressive functions [37]. The C2/EAR motif is important for maintaining the inhibitory function of the repressors. Earlier studies proved that this motif can convert transcriptional activators into repressors, and that the complete or partial deletion of the EAR sequence will diminish or eliminate the repressive activity of a repressor [38–40]. In addition to the C2/EAR motif, TLLFR is another repressor motif. This motif was first detected in AtMYBL2, which is a strong inhibitor of flavonoid synthesis [41]. Cavallini *et al*. [42] reported that the R2R3-MYB TFs MYBC2-L1, MYBC2-L2, and MYBC2-L3 also contain TLLFR motifs, which may explain their strong repressive effects on anthocyanin synthesis. The MdMYB17 examined in this study contains the LxLxL C2/EAR motif, but not the TLLFR motif. The transgenic assays combined with promoter activity analyses revealed that MdMYB17 directly represses the expression of anthocyanin synthesis-related genes. Moreover, deleting the C2/EAR motif diminishes the repressive activity of MdMYB17, reflecting the importance of the C2/EAR motif for MdMYB17 functionality. Additionally, our findings indicate that the inhibitory function of the C2/EAR motif is conserved.

### There is a complex regulatory relationship between *MdMYB17* and *MdMYB1*

There is evidence suggesting that anthocyanin biosynthesis is jointly regulated by the R2R3-MYB activators and repressors. For example, *PpMYB18*, which encodes an R2R3-MYB TF that represses anthocyanin synthesis, is co-expressed with *PpMYB10.1*, which encodes an anthocyanin synthesis activator, during peach fruit coloration; the expression of *PpMYB18* is induced by PpMYB10.1 [30]. In strawberry, the *FcMYB1* expression level is highest when the anthocyanins accumulate to their peak levels during the fruit coloration period [43]. In the present study, we determined that the expression levels of *MdMYB17* (R2R3-MYB TF that represses anthocyanin synthesis) and *MdMYB1* (R2R3-MYB TF that activates anthocyanin synthesis) were up-regulated by ethylene during the fruit coloration stage. This implies that anthocyanin synthesis in apple fruit is regulated by MdMYB17 and MdMYB1. The results of our promoter binding assay and LUC assay revealed that MdMYB17 represses *MdMYB1* transcription, whereas MdMYB1 induces *MdMYB17* transcription. Thus, a feedback regulatory loop between MdMYB1 and MdMYB17 controls the expression of the corresponding genes. The dynamic balance between *MdMYB1* and *MdMYB17* expression levels and their regulatory effects on anthocyanin synthesis are maintained by this mechanism.

In this study, we found that ethylene induces the expression of R2R3-MYB repressor *MdMYB17*, but it also strongly promotes apple anthocyanin synthesis. Additionally, ethylene induces R2R3-MYB activator *MdMYB1* expression. Similarly, ethephon treatments can up-regulate the expression of the R2R3-MYB repressor *PpMYB140* in pear. However, ethephon treatments inhibited the accumulation of pear fruit anthocyanins and down-regulated the expression of the R2R3-MYB activators *PpMYB10* and *PpMYB114* [24]. These results suggest that the changes in the anthocyanin contents of apple and pear fruits treated with ethephon are consistent with the expression of the R2R3-MYB activator, but not the R2R3-MYB repressor. It is possible that the effect of the R2R3-MYB activator’s activation on anthocyanin synthesis exceeds that of the repression effect of R2R3-MYB repressor in fruits.

### The mechanism underlying the *MdMYB17* response to ethylene is complex

The mechanism underlying the response of the R2R3-MYB repressor to ethylene is unclear. In this study, the ethephon treatment up-regulated the *MdMYB17* expression level via the ethylene signal transducer *MdEIL1*, indicating that the inhibitory effect of *MdMYB17* on anthocyanin production is regulated by ethylene. Anthocyanins have important functions related to plant resistance to diseases and UV irradiation [2, 3], but the excessive accumulation of anthocyanins may lead to an imbalance in the primary and secondary metabolic activities of plants [44]. Ethylene can strongly induce anthocyanin synthesis. We speculate that the up-regulated expression of *MdMYB17* can prevent the excessive accumulation of anthocyanins and maintain ethylene-induced apple anthocyanin synthesis at an appropriate level.

The ethylene receptor inhibitor 1-MCP can disrupt ethylene signal transduction to minimize the physiological effect of ethylene (e.g. inhibition of anthocyanin synthesis). An *et al*. [22] reported that an ethephon treatment up-regulates the *MdEIL1* and *MdMYB1* expression levels and promotes anthocyanin synthesis in apple. In contrast, a 1-MCP treatment inhibits the expression of *MdEIL1* and *MdMYB1* and anthocyanin synthesis. Our findings are consistent with those of An *et al*. [22]. However, the 1-MCP treatment up-regulated *MdMYB17* expression, but had the opposite effect on *MdEIL1* expression. Therefore, the mechanism by which ethylene up-regulates the expression of *MdMYB17* through *MdEIL1* cannot explain why 1-MCP up-regulates the expression of *MdMYB17*. Additionally, Ni *et al*. [24] also observed that 1-MCP up-regulates the expression of the R2R3-MYB repressor *PpMYB140* in ‘Hongzaosu’ pear. These results indicate that 1-MCP up-regulating the expression of the R2R3-MYB repressor may be a common phenomenon in fruits. However, the underlying molecular mechanism remains to be characterized. We speculate that although 1-MCP decreases the *MdEIL1*-mediated up-regulated expression of *MdMYB17*, it may modulate the expression of other regulatory genes, which may lead to the up-regulated expression of *MdMYB17*.

### Relationship between MdMYB17 and MdEIL1 during ethylene-regulated anthocyanin synthesis and fruit coloration in apple

Ethylene is crucial for regulating anthocyanin accumulation and coloration in apple fruit. An *et al*. [22] investigated the molecular basis of ethylene-induced anthocyanin synthesis in apple. They reported that MdEIL1 activates *MdMYB1* expression and ethylene promotes anthocyanin synthesis and apple fruit coloration through the MdEIL1–MdMYB1 signaling pathway. In the present study, we observed that MdEIL1 binds directly to the *MdMYB17* promoter to activate expression, indicating that ethylene also inhibits anthocyanin synthesis through the MdEIL1–MdMYB17 signaling pathway. It is likely that MdEIL1 positively and negatively affects ethylene-regulated anthocyanin synthesis in apple. We also confirmed that MdMYB17 represses the expression of *MdEIL1* by directly binding to its promoter. Therefore, the relationship between MdEIL1 and MdMYB17 probably influences the regulatory effect of MdEIL1 on anthocyanin synthesis.

Previous studies proved that MdMYB1 does not interact with MdEIL1 [22]. However, in the present study, we revealed that MdMYB17 interacts with MdEIL1, indicative of a direct regulatory relationship between MdEIL1 and the anthocyanin synthesis-related R2R3-MYB TFs at the protein level. LUC reporter assays indicated that the co-expression of MdMYB17 and MdEIL1 proteins attenuates the negative effects of MdMYB17 on the *MdEIL1* and *MdMYB1* promoters and the positive effects of MdEIL1 on the *MdMYB1* and *MdMYB17* promoters. Accordingly, the interaction between MdMYB17 and MdEIL1 is critical for maintaining the *MdEIL1*, *MdMYB17*, and *MdMYB1* expression levels as well as anthocyanin synthesis.

**Figure 8:**
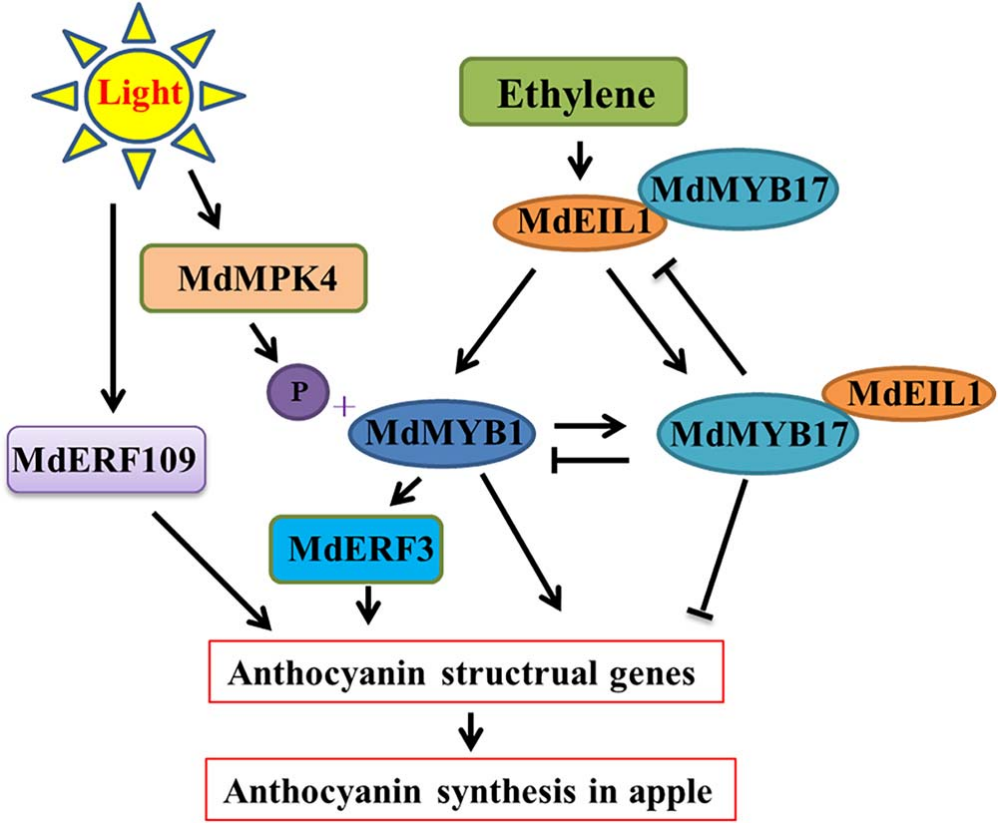
Proposed model of ethylene-regulated anthocyanin synthesis in apple fruit. MdEIL1 directly activates *MdMYB1* expression to increase structural gene expression and anthocyanin accumulation. Additionally, MdEIL1 directly activates *MdMYB17* expression to inhibit structural gene expression and anthocyanin accumulation. In contrast, MdMYB17 directly represses *MdMYB1* and *MdEIL1* expression to inhibit anthocyanin accumulation. Furthermore, MdMYB17 and MdEIL1 form a protein complex that down-regulates the effects of MdMYB17 on *MdMYB1* and *MdEIL1* as well as the regulatory effects of MdEIL1 on *MdMYB17*. In addition, MdMYB1 directly activates *MdERF3* expression [22] to increase anthocyanin accumulation [45]. Light can promote anthocyanin accumulation by increasing the expression of *MdERF109* [47] and the production of the phosphorylated MdMYB1 protein [46].

On the basis of the data presented herein, we propose the following working model for the roles of *MdEIL1*, *MdMYB17*, and *MdMYB1* related to ethylene-regulated anthocyanin synthesis in apple fruits (Fig. 8). In this model, the initiation of apple fruit maturation activates the ethylene signal. Additionally, the *MdEIL1–MdMYB1* regulatory pathway, which promotes anthocyanin synthesis, and the *MdEIL1–MdMYB17* regulatory pathway, which inhibits anthocyanin synthesis, are induced. The activation of *MdMYB17* expression by MdMYB1 and MdEIL1 results in the feedback regulation of *MdEIL1* and *MdMYB1* expression. At the same time, the MdMYB17–MdEIL1 protein interaction weakens the regulatory effects of MdMYB17 on *MdMYB1* and *MdEIL1* and of MdEIL1 on *MdMYB17* and *MdMYB1*. Moreover, some studies revealed that MdMYB1 activates *MdERF3* expression, which can promote anthocyanin synthesis [22, 45]. In response to light, MdMYB1 is phosphorylated by the light-induced MdMPK4, which stabilizes the TF and promotes anthocyanin synthesis [46]. Light also increases anthocyanin synthesis by inducing the expression of the ethylene signal transducer *MdERF109* [47]. These findings indicate that the regulation of anthocyanin synthesis in apple fruits involves many genes that respond quickly to developmental and environmental signals.

## Materials and methods

### Plant materials and treatments

*Malus domestica* cv. ‘Geneva Early’ fruits were harvested at 60 DAFB and divided into three groups. The first group was left untreated (i.e. control). The second group was treated with 1000 mg l^−1^ ethephon solution for 1 min. The third group was treated with 1 μl l^−1^ 1-MCP for 12 h. All fruits were then stored at room temperature (24°C) and exposed to constant light (20 000 lux) for 6 days. The fruit anthocyanin content and ethylene production were measured at 0, 3, and 6 days during the storage period. Three fruits were used as a biological replicate and three biological replicates were set at each sampling time-point for the subsequent measurements.

Apple fruits (cv. ‘Yinv’) were harvested at 50 and 140 DAFB for injection assays. ‘Orin’ apple calli were used for *Agrobacterium tumefaciens*-mediated transformation.

### Determination of anthocyanin contents and ethylene production

Anthocyanin contents were measured according to the pH differential method [48]. Briefly, 1 g powdered apple peel was mixed with 10 ml 1% (v/v) HCl–methanol at 4°C for 24 h. Then, 1 ml supernatant was added to 4 ml KCl buffer (pH 1.0) and 4 ml NaAc buffer (pH 4.5). The absorbance (at 510 and 700 nm) of the mixture was determined using a UV-2450 spectrophotometer (Shimadzu, Kyoto, Japan).

Ethylene production was analyzed using the Clarus 580 GC system (PerkinElmer, Waltham, MA, USA) as previously described [32, 49].

### Quantitative PCR

Total RNA was extracted, cDNA was synthesized, and a qPCR analysis was performed as previously described [50]. The iCycler iQ5 system (Bio-Rad Laboratories, CA, USA) was used for the qPCR. MdActin was used as the internal control, and the expression levels were calculated using the 2^−△△Ct^ method [51]. Details regarding the qPCR primers are provided in Supplementary Data Table S1.

### Generation of transgenic apple calli

The full-length *MdMYB1*, *MdMYB17*, and *MdEIL1* CDSs were inserted into the pRI101-AN vector containing a GFP tag to obtain the overexpression vectors *MdMYB17*-pRI, *MdMYB1*-pRI, and *MdEIL1*-pRI. Additionally, 390-bp sense and antisense sequences of the *MdMYB17* CDS were inserted into the pFGC1008 vector to obtain the RNAi construct *MdMYB17*-RNAi. The recombinant plasmids were inserted into *A. tumefaciens* strain LBA4404 cells. Analyses of the overexpression of *MdMYB17*, *MdMYB1*, and *MdEIL1* and the silencing of *MdMYB17* in apple calli were performed as previously described [52]. Each successfully transformed calli line was used as a biological replicate. Three biological replicates were analyzed for the gene overexpression or RNAi assays.

### Fruit injection assay

Fruit injection assays were performed as previously described [32]. A 390 bp sequence of the *MdMYB17* CDS was inserted into the tobacco rattle virus (TRV) vector to obtain the antisense virus vector MdMYB17-TRV [53]. The overexpression vector MdMYB17-pRI was constructed as mentioned above. The recombinant plasmids were inserted into *A. tumefaciens* strain LBA4404 cells. The vectors and the *A. tumefaciens* solutions were injected into apple fruit peels. The injected fruits were stored at 24°C and exposed to constant light (20 000 lux) for 5 days. Ten injected fruits were used as a biological replicate. Each fruit injection assay was performed using three biological replicates.

### Yeast one-hybrid assay

The full-length *MdMYB17*, *MdMYB1*, and *MdEIL1* CDSs were inserted into the pGADT7 vector to obtain the recombinant vectors MdMYB17-pGAD, MdMYB1-pGAD, and MdEIL1-pGAD. The *MdMYB17*, *MdMYB1*, *MdDFR*, *MdUFGT*, and *MdEIL1* promoter fragments were inserted into the pHIS2 vector. Yeast strain Y187 cells harboring the recombinant pHIS2 plasmids were cultured on SD/−Trp/−Leu/−His medium containing 3-amino-1,2,4-triazole (3-AT).

### Electrophoretic mobility shift assay

The full-length *MdMYB17*, *MdMYB1*, and *MdEIL1* CDSs were inserted into the pET-32a vector containing a His tag to obtain the recombinant plasmids, which were then inserted into *Escherichia coli* BL21 (DE3) cells for the production of fusion proteins. The fusion proteins were purified using the His-tagged Protein Purification Kit (CWbio, Beijing, China). The EMSAs were conducted using the LightShift Chemiluminescent EMSA kit (Thermo Scientific, Waltham, MA, USA). All probes were synthesized by Sangon Biotechnology Co., Ltd. (Shanghai, China).

### Chromatin immunoprecipitation–quantitative PCR analysis

Transgenic calli harboring *MdMYB1*, *MdMYB17*, and *MdEIL1* fused to a GFP tag were obtained as described above. The ChIP assays were performed using the EZ-ChIP Chromatin Immunoprecipitation Kit (Millipore/Upstate, MA, USA) as previously described [52]. Apple callus overexpressing the GFP sequence was used as a control. The enriched DNA fragments were examined by qPCR. The primers are provided in Supplementary Data Table S1.

### Luciferase analysis

The LUC analysis was performed as previously described [52]. The full-length *MdMYB1*, *MdMYB17*, and *MdEIL1* CDSs were inserted into pHBT-AvrRpm1 to obtain the effectors. The *MdMYB1*, *MdMYB17*, *MdDFR*, *MdUFGT*, and *MdEIL1* promoter fragments were inserted into pFRK1-LUC-nos to obtain the reporters. Protoplasts were prepared from ‘Orin’ apple calli. The LUC activity values were recorded using the Victor X4 Multimode Plate Reader (PerkinElmer, Norwalk, CT, USA).

### Yeast two-hybrid assay

The full-length *MdMYB17* CDS was inserted into the pGBKT7 vector, whereas the full-length *MdEIL1* CDS was inserted into the pGADT7 vector. Cells of the Y2HGold yeast strain harboring the recombinant plasmids were cultured on SD/−Trp/−Leu/−His/−Ade medium containing X-α-gal.

### Bimolecular fluorescence complementation assay

The BiFC assay was performed as previously described [50]. The full-length *MdMYB17* CDS without the stop codon was inserted into the pSPYNE-35S vector, whereas the *MdEIL1* CDS was inserted into the pSPYCE-35S vector. The recombinant plasmids were inserted into *A. tumefaciens* LBA4404 cells. The YFP signals were detected using a DS-Ri2 confocal laser scanning microscope (Nikon Corporation, Tokyo, Japan) at an excitation wavelength of 488 nm.

### Pull-down assay

The full-length *MdMYB17* CDS was inserted into the pET-32a(+) vector containing a His tag, whereas the *MdEIL1* CDS was inserted into the pGEX-4 T-1 vector containing a GST tag. The recombinant plasmids were inserted into BL21 cells to produce fusion proteins. The MdEIL1-GST protein was incubated with MdMYB17-HIS protein or His-tagged bait protein. Proteins were eluted in elution buffer and then analyzed in an immunoblot using anti-His and anti-GST antibodies (Abmart, Shanghai, China).

### Co-immunoprecipitation assay

The Co-IP assay was performed as previously described [54]. The full-length *MdMYB17* CDS was inserted into the pHBT-AvrRpm1-HA vector containing an HA tag, whereas the *MdEIL1* CDS was inserted into the pHBT-AvrRpm1-FLAG vector containing a FLAG tag. The MdMYB17-HA and MdEIL1-FLAG constructs were used for the co-transfection of ‘Orin’ protoplasts prepared from apple calli. After centrifugation, the supernatant was mixed with anti-FLAG agarose beads. A western blot analysis was performed using anti-HA and anti-FLAG antibodies.

## Acknowledgements

We thank Liwen Bianji (Edanz) (www.liwenbianji.cn/) for editing the English text of a draft of this manuscript. We thank Aide Wang and Tong Li (Shenyang Agricultural University), and Jianping An (Shandong Agricultural University) for their help in this work. This work was supported by the National Natural Science Foundation of China (31872940), the National Key Research and Development Program of China (2018YFD1000105), and the Agricultural Improved Seed Project of Shandong Province (2019LZGC008).

## Author contributions

S.-Q.F. designed the research. S.W., L.-X.L., Z.Z., Y.F. and D.L. performed the experiments, conducted fieldwork and analyzed the data. S.-Q.F. and X.-S.C. analyzed the data. S.-Q.F. wrote the manuscript.

## Data availability

All data supporting the findings of this study are available within the paper and within its supplementary data.

## Conflict of interest

None declared.

## Supplementary data

Supplementary data is available at *Horticulture Research* online.

## Supplementary Material

Web_Material_uhac034

## References

[1] Cipollini ML , Levey DJ. Secondary metabolites of fleshy vertebrate-dispersed fruits: adaptive hypotheses and implications for seed dispersal. Am Nat. 1997;150:346–72. 18811294 10.1086/286069

[2] Gould KS . Nature's Swiss army knife: the diverse protective roles of anthocyanins in leaves. J Biomed Biotechnol. 2004;5:314–20. 10.1155/S1110724304406147 PMC1082902 15577195

[3] Schaefer HM , Schaefer V, Levey DJ. How plant-animal interactions signal new insights in communication. Trends Ecol Evol. 2004;19:577–84.

[4] Espley RV , Hellens RP, Jo P et al. Red colouration in apple fruit is due to the activity of the MYB transcription factor, MdMYB10. Plant J. 2007;49:414–27. 17181777 10.1111/j.1365-313X.2006.02964.x PMC1865000

[5] Tanaka Y , Sasaki N, Ohmiya A. Biosynthesis of plant pigments: anthocyanins, betalains and carotenoids. Plant J. 2008;54:733–49. 18476875 10.1111/j.1365-313X.2008.03447.x

[6] Koes R , Verweij W, Quattrocchio F. Flavonoids: a colorful model for the regulation and evolution of biochemical pathways. Trends Plant Sci. 2005;10:236–42. 15882656 10.1016/j.tplants.2005.03.002

[7] Gonzalez A , Zhao MZ, Leavitt JM et al. Regulation of the anthocyanin biosynthetic pathway by the TTG1/bHLH/Myb transcriptional complex in *Arabidopsis* seedlings. Plant J. 2008;53:814–27. 18036197 10.1111/j.1365-313X.2007.03373.x

[8] Hichri I , Barrieu F, Bogs J et al. Recent advances in the transcriptional regulation of the flavonoid biosynthetic pathway. J Exp Bot. 2011;62:2465–83. 21278228 10.1093/jxb/erq442

[9] Kranz HD , Denekamp M, Greco R et al. Towards functional characterisation of the members of the R2R3-MYB gene family from *Arabidopsis thaliana*. Plant J. 1998;16:263–76. 9839469 10.1046/j.1365-313x.1998.00278.x

[10] Stracke R , Werber M, Weisshaar B. The R2R3–MYB gene family in *Arabidopsis thaliana*. Curr Opin Plant Biol. 2001;l4:447–56. 10.1016/s1369-5266(00)00199-0 11597504

[11] Yanhui C , Xiaoyuan Y, Kun H et al. The MYB transcription factor superfamily of *Arabidopsis*: expression analysis and phylogenetic comparison with the rice MYB family. Plant Mol Biol. 2006;60:107–24. 16463103 10.1007/s11103-005-2910-y

[12] Dubos C , Stracke R, Grotewold E et al. MYB transcription factors in *Arabidopsis*. Trends Plant Sci. 2010;15:573–81. 20674465 10.1016/j.tplants.2010.06.005

[13] Liu YH , Lin-Wang K, Espley RV et al. StMYB44 negatively regulates anthocyanin biosynthesis at high temperatures in tuber flesh of potato. J Exp Bot. 2019;70:3809–24. 31020330 10.1093/jxb/erz194 PMC6685667

[14] Chen LH , Hu B, Qin YH et al. Advance of the negative regulation of anthocyanin biosynthesis by MYB transcription factors. Plant Physiol Biochem. 2019;136:178–87. 30685697 10.1016/j.plaphy.2019.01.024

[15] Ban Y , Honda C, Hatsuyama Y et al. Isolation and functional analysis of a MYB transcription factor gene that is a key regulator for the development of red coloration in apple skin. Plant Cell Physiol. 2007;48:958–70. 17526919 10.1093/pcp/pcm066

[16] Lin-Wang K , Micheletti D, Palmer J et al. High temperature reduces apple fruit colour via modulation of the anthocyanin regulatory complex. Plant Cell Environ. 2011;34:1176–90. 21410713 10.1111/j.1365-3040.2011.02316.x

[17] Xu HF , Wang N, Liu JX et al. The molecular mechanism underlying anthocyanin metabolism in apple using the MdMYB16 and MdbHLH33 genes. Plant Mol Biol. 2017;94:149–65. 28286910 10.1007/s11103-017-0601-0

[18] Rubin G , Tohge T, Matsuda F et al. Members of the LBD family of transcription factors repress anthocyanin synthesis and affect additional nitrogen responses in *Arabidopsis*. Plant Cell. 2009;21:3567–84. 19933203 10.1105/tpc.109.067041 PMC2798321

[19] Takos AM , Jaffé FW, Jacob SR et al. Light induced expression of a MYB gene regulates anthocyanin biosynthesis in red apples. Plant Physiol. 2006;142:1216–32. 17012405 10.1104/pp.106.088104 PMC1630764

[20] Whale SK , Singh Z. Endogenous ethylene and color development in the skin of ‘pink lady’ apple. J Am Soc Hortic Sci. 2007;132:20–8.

[21] An XH , Tian Y, Chen KQ et al. MdMYB9 and MdMYB11 are involved in the regulation of the JA-induced biosynthesis of anthocyanin and proanthocyanidin in apples. Plant Cell Physiol. 2015;56:650–62. 25527830 10.1093/pcp/pcu205

[22] An JP , Wang XF, Li YY et al. EIN3-LIKE1, MYB1, and ETHYLENE RESPONSE FACTOR3 act in a regulatory loop that synergistically modulates ethylene biosynthesis and anthocyanin accumulation. Plant Physiol. 2018;178:808–23. 29925585 10.1104/pp.18.00068 PMC6181056

[23] An JP , Wang XF, Zhang XW et al. An apple MYB transcription factor regulates cold tolerance and anthocyanin accumulation and undergoes MIEL1-mediated degradation. Plant Biotechnol J. 2020;18:337–53. 31250952 10.1111/pbi.13201 PMC6953192

[24] Ni JB , Premathilake AT, Gao YH et al. Ethylene-activated PpERF105 induces the expression of the repressor-type R2R3-MYB gene PpMYB140 to inhibit anthocyanin biosynthesis in red pear fruit. Plant J. 2021;105:167–81. 33111423 10.1111/tpj.15049

[25] Klee HJ , Giovannoni JJ. Genetics and control of tomato fruit ripening and quality attributes. Annu Rev Genet. 2011;45:41–59. 22060040 10.1146/annurev-genet-110410-132507

[26] Giovannoni JJ . Genetic regulation of fruit development and ripening. Plant Cell. 2004;16:S170–80. 15010516 10.1105/tpc.019158 PMC2643394

[27] Wang KL , Li H, Ecker JR. Ethylene biosynthesis and signaling networks. Plant Cell. 2002;14:S131–51. 12045274 10.1105/tpc.001768 PMC151252

[28] Wang LK , Zhang ZY, Zhang F et al. EIN2-directed histone acetylation requires EIN3-mediated positive feedback regulation in response to ethylene. Plant Cell. 2021;33:322–37. 33793786 10.1093/plcell/koaa029 PMC8136887

[29] Espley RV , Leif D, Plunkett B et al. Red to brown: an elevated anthocyanic response in apple drives ethylene to advance maturity and fruit flesh browning. Front Plant Sci. 2019;10:1248. 31649709 10.3389/fpls.2019.01248 PMC6794385

[30] Zhou H , Lin-Wang K, Wang FR et al. Activator-type R2R3-MYB genes induce a repressor-type R2R3-MYB gene to balance anthocyanin and proanthocyanidin accumulation. New Phytol. 2019;221:1919–34. 30222199 10.1111/nph.15486

[31] Song J , Bangerth F. The effect of harvest date on aroma compound production from ‘Golden Delicious’ apple fruit and relationship to respiration and ethylene production. Postharvest Biol Technol. 1996;8:259–69.

[32] Li T , Jiang ZY, Zhang LC et al. Apple (*Malus domestica*) MdERF2 negatively affects ethylene biosynthesis during fruit ripening by suppressing *MdACS1* transcription. Plant J. 2016;88:735–48. 27476697 10.1111/tpj.13289

[33] Li T , Xu YX, Zhang LC et al. The jasmonate-activated transcription factor MdMYC2 regulates *ETHYLENE RESPONSE FACTOR* and ethylene biosynthetic genes to promote ethylene biosynthesis during apple fruit ripening. Plant Cell. 2017;29:1316–34. 28550149 10.1105/tpc.17.00349 PMC5502464

[34] Zhu ZQ , An FY, Feng Y et al. Derepression of ethylene-stabilized transcription factors (EIN3/EIL1) mediates jasmonate and ethylene signaling synergy in *Arabidopsis*. Proc Natl Acad Sci USA. 2011;108:12539–44. 21737749 10.1073/pnas.1103959108 PMC3145709

[35] Lingam S , Mohrbacher J, Brumbarova T et al. Interaction between the bHLH transcription factor FIT and ETHYLENE INSENSITIVE3/ETHYLENE INSENSITIVE3-LIKE1 reveals molecular linkage between the regulation of iron acquisition and ethylene signaling in *Arabidopsis*. Plant Cell. 2011;23:1815–29. 21586684 10.1105/tpc.111.084715 PMC3123957

[36] Yao GF , Ming ML, Allan AC et al. Map-based cloning of the pear gene MYB114 identifies an interaction with other transcription factors to coordinately regulate fruit anthocyanin biosynthesis. Plant J. 2017;92:437–51. 28845529 10.1111/tpj.13666

[37] Kagale S , Links MG, Rozwadowski K. Genome-wide analysis of ethylene-responsive element binding factor-associated amphiphilic repression motif-containing transcriptional regulators in *Arabidopsis*. Plant Physiol. 2010;152:1109–34. 20097792 10.1104/pp.109.151704 PMC2832246

[38] Albert NW , Davies KM, Lewis DH et al. A conserved network of transcriptional activators and repressors regulates anthocyanin pigmentation in eudicots. Plant Cell. 2014;26:962–80. 24642943 10.1105/tpc.113.122069 PMC4001404

[39] Wan SZ , Li CF, Ma XD et al. PtrMYb57 contributes to the negative regulation of anthocyanin and proanthocyanidin biosynthesis in poplar. Plant Cell Rep. 2017;36:1263–76. 28523445 10.1007/s00299-017-2151-y

[40] Jun JH , Liu CG, Xiao XR et al. The transcriptional repressor MYB2 regulates both spatial and temporal patterns of proanthocyandin and anthocyanin pigmentation in *Medicago truncatula*. Plant Cell. 2015;27:2860–79. 26410301 10.1105/tpc.15.00476 PMC4682322

[41] Matsui K , Umemura Y, Ohme-Takagi M. AtMYBL2, a protein with a single MYB domain, acts as a negative regulator of anthocyanin biosynthesis in *Arabidopsis*. Plant J. 2008;55:954–67. 18532977 10.1111/j.1365-313X.2008.03565.x

[42] Cavallini E , Matus JT, Finezzo L et al. The phenylpropanoid pathway is controlled at different branches by a set of R2R3-MYB C2 repressors in grapevine. Plant Physiol. 2015;167:1448–70. 25659381 10.1104/pp.114.256172 PMC4378173

[43] Salvatierra A , Pimentel P, Moya-León MA et al. Increased accumulation of anthocyanins in *Fragaria chiloensis* fruits by transient suppression of *FcMYB1* gene. Phytochemistry. 2013;90:25–36. 23522932 10.1016/j.phytochem.2013.02.016

[44] Jeong SW , Das PK, Jeoung SC et al. Ethylene suppression of sugar-induced anthocyanin pigmentation in *Arabidopsis*. Plant Physiol. 2010;154:1514–31. 20876338 10.1104/pp.110.161869 PMC2971625

[45] Bi SQ , An JP, Wang XF et al. Ethylene response factor MdERF3 promotes anthocyanin and proanthocyanidin accumulation in apple. Acta Horticulturae Sinica. 2019;46:2277–85.

[46] Yang T , Ma HY, Li Y et al. Apple MPK4 mediates phosphorylation of MYB1 to enhance light-induced anthocyanin accumulation. Plant J. 2021;106:1728–45. 33835607 10.1111/tpj.15267

[47] Ma HY , Yang T, Li Y et al. The long noncoding RNA MdLNC499 bridges MdWRKY1 and MdERF109 function to regulate early-stage light-induced anthocyanin accumulation in apple fruit. Plant Cell. 2021;33:3309–30. 34270784 10.1093/plcell/koab188 PMC8505877

[48] Giusti MM , Wrolstad RE. Characterization and measurement of anthocyanins by UV-visible spectroscopy. In: Wrolstad RE (ed.), *Current Protocols in Food Analytical Chemistry*. New York: Wiley, 2001, F1.2.1–13.

[49] Li TZ , Tan DM, Yang X et al. Apple 1-aminocyclopropane-1-carboxylic acid synthase genes, *MdACS1* and *MdACS3a*, are expressed in different systems of ethylene biosynthesis. Plant Mol Biol. 2013;31:204–9.

[50] Feng SQ , Sun SS, Chen XL et al. PyMYB10 and PyMYB10.1 interact with bHLH to enhance anthocyanin accumulation in pears. PLoS One. 2015;10:e0142112. 26536358 10.1371/journal.pone.0142112 PMC4633228

[51] Livak KJ , Schmittgen TD. Analysis of relative gene expression data using real-time quantitative PCR and the 2(-delta delta C(T)) method. Methods. 2001;25:402–8. 11846609 10.1006/meth.2001.1262

[52] Wang N , Qu CZ, Jiang SH et al. The proanthocyanidin-specific transcription factor MdMYBPA1 initiates anthocyanin synthesis under low-temperature conditions in red-fleshed apples. Plant J. 2018;96:39–55. 29978604 10.1111/tpj.14013

[53] Dinesh-Kumar SP , Anandalakshmi R, Marathe R et al. Virus-induced gene silencing. Methods Mol Biol. 2003;236:287–94. 14501071 10.1385/1-59259-413-1:287

[54] Wang N , Xu HF, Jiang SH et al. MYB12 and MYB22 play essential roles in proanthocyanidin and flavonol synthesis in red-fleshed apple (*Malus sieversii* f. *niedzwetzkyana*). Plant J. 2017;90:276–92. 28107780 10.1111/tpj.13487

