# Supplementary material for: Ethylene precisely regulates anthocyanin synthesis in apple via a module comprising MdEIL1, MdMYB1, and MdMYB17
Source: Hortic Res. 2022 Feb 19;9:uhac034. doi: 10.1093/hr/uhac034 (PMC9039505; doi:10.1093/hr/uhac034)
Supplement: Web_Material_uhac034 [file web_material_uhac034.zip › supplementary information.docx]

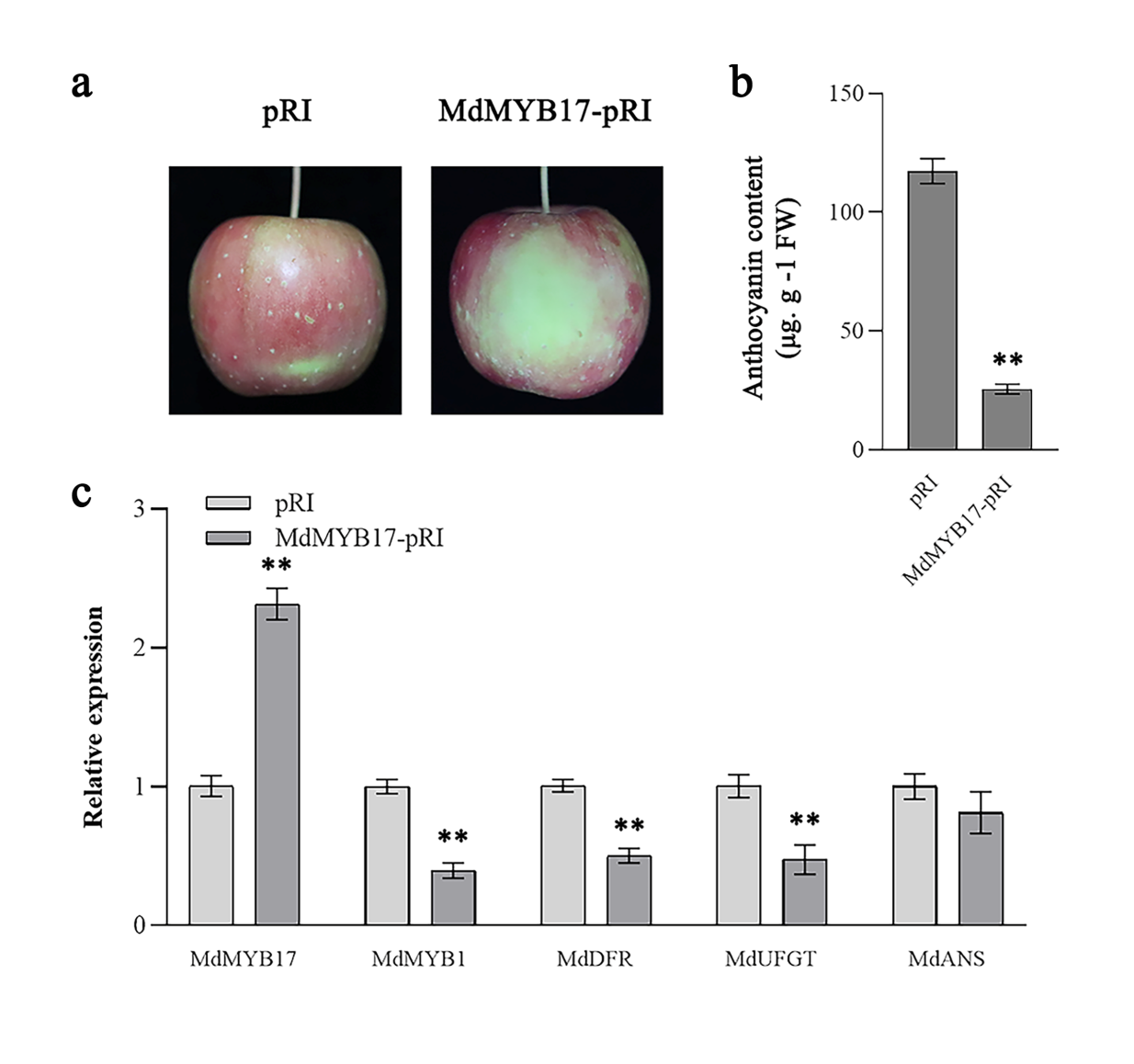


**Fig. S1:** Transient overexpression of *MdMYB17* inhibits anthocyanin synthesis in apple fruit. **a** Phenotypes of ‘Yinv’ apple fruit transiently overexpressing *MdMYB17*. **b** Anthocyanin contents of the fruit pericarp around the injection sites. FW, fresh weight. **c** Expression levels of *MdMYB17* and anthocyanin-related genes in the fruit pericarp around the injection sites. Apple fruit were collected at 140 DAFB. Data are presented as the mean ± SD of three independent biological replicates. Asterisks indicate significant differences, as determined by Student’s *t*-test (**P* < 0.05, ***P* < 0.01).


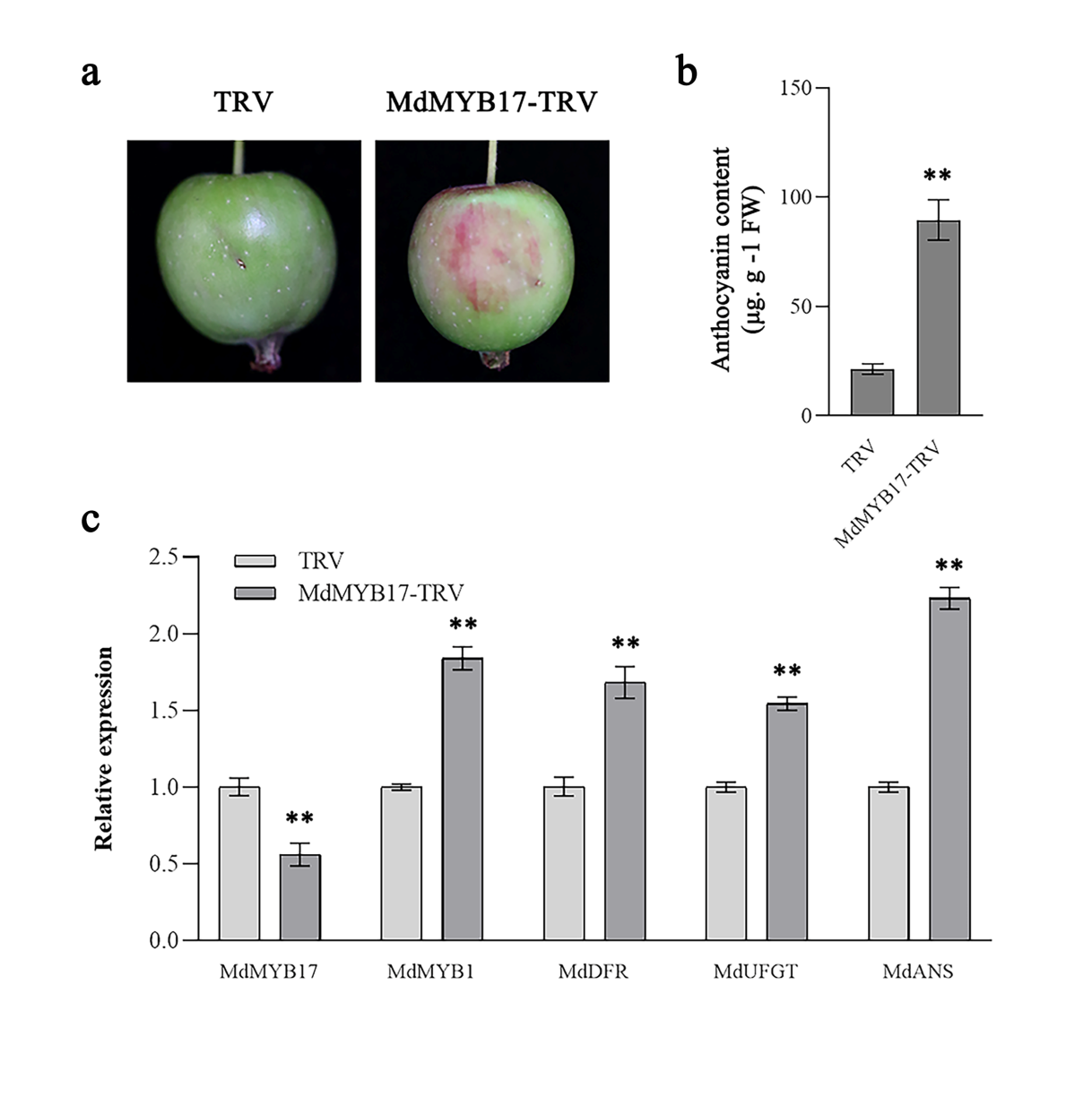


**Fig. S2:** Transient silencing of *MdMYB17* induces anthocyanin synthesis in apple fruit. **a** Phenotypes of ‘Yinv’ apple fruit in which *MdMYB17* is transiently silenced. **b** Anthocyanin contents of the fruit pericarp around the injection sites. FW, fresh weight. **c** Expression levels of *MdMYB17* and anthocyanin-related genes in the fruit pericarp around the injection sites. Apple fruit were collected at 50 DAFB. Data are presented as the mean ± SD of three independent biological replicates. Asterisks indicate significant differences, as determined by Student’s *t*-test (**P* < 0.05, ***P* < 0.01).


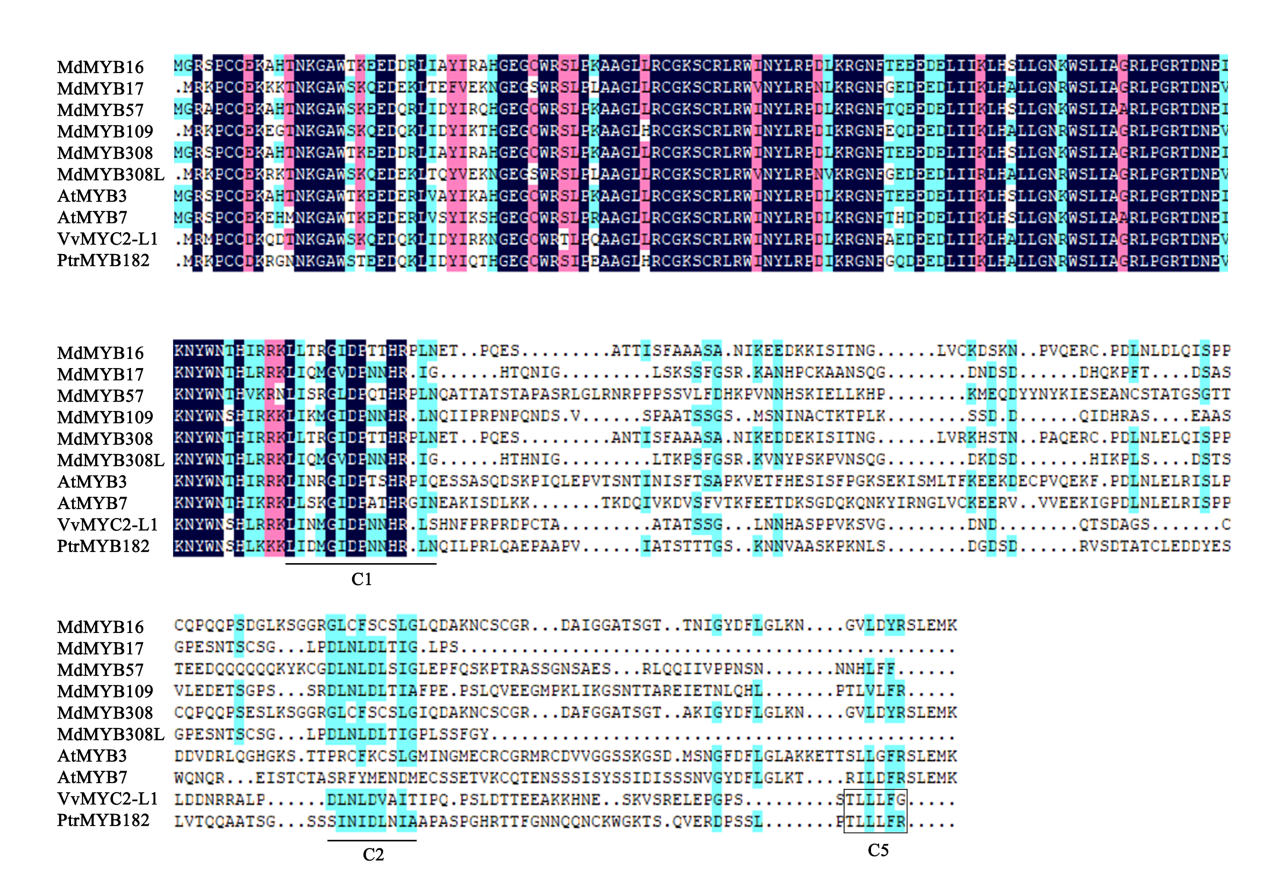
**Fig. S3:** Protein sequence alignment of MdMYB17 and subfamily 4 R2R3-MYB TFs. The conserved C2/EAR and C5/TLLFR motifs are underlined or boxed. GenBank accession numbers are listed in Supplemental Table 2.


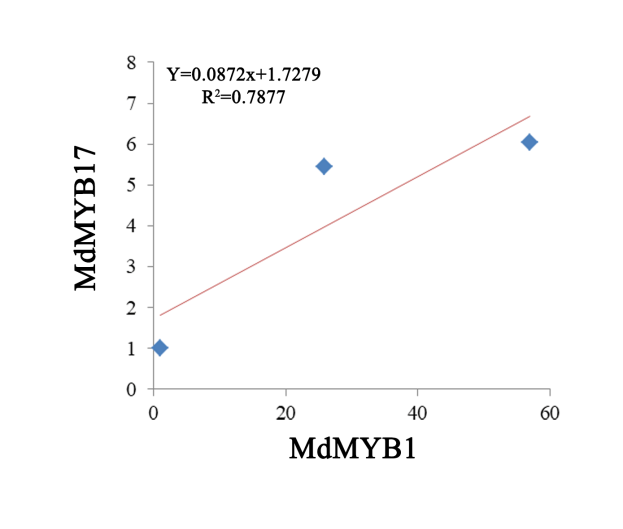


**Fig. S4:** *MdMYB1* and *MdMYB17* expression levels are positively correlated (R^2^ = 0.7877). Correlation coefficients were calculated using Excel (Han *et al.,* 2016).

Han, B.Y., Fu, L.L., Zhang, D., He, X.Q., Chen, Q., Peng, M. & Zhang, J.M. Interspecies and intraspecies analysis of trehalose contents and the biosynthesis pathway gene family reveals crucial roles of trehalose in osmotic-stress tolerance in cassava. *Int. J. Mol. Sci.* **17(7),** 1077 (2016).


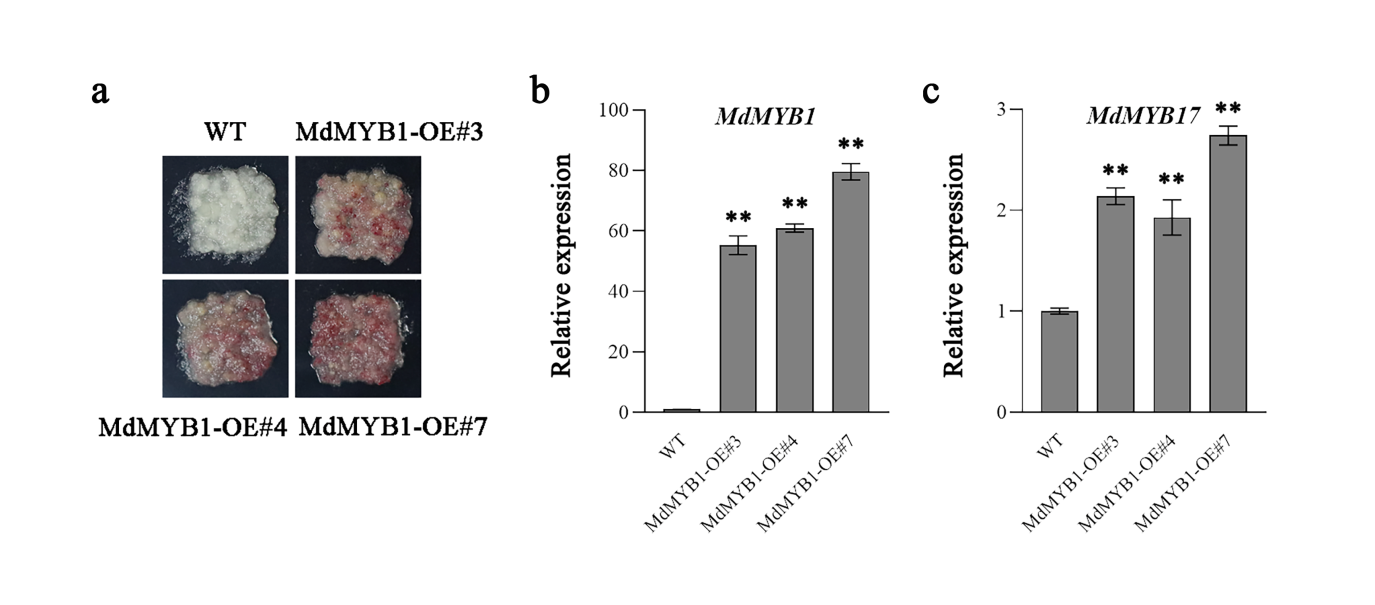
**Fig. S5:** MdMYB1 activates anthocyanin synthesis and *MdMYB17* expression in ‘Orin’ apple calli. **a** Phenotypes of ‘Orin’ apple calli incubated at 14 °C and light（20,000 lux） for 10 days. WT: wild-type; *MdMYB1*-OE: *MdMYB1*-overexpression. **(b and c)** Expression levels of *MdMYB1* **(b)** and *MdMYB17* **(c)** in apple calli. Data are presented as the mean ± SD of three independent biological replicates. Asterisks indicate significant differences, as determined by Student’s *t*-test (**P* < 0.05, ***P* < 0.01).


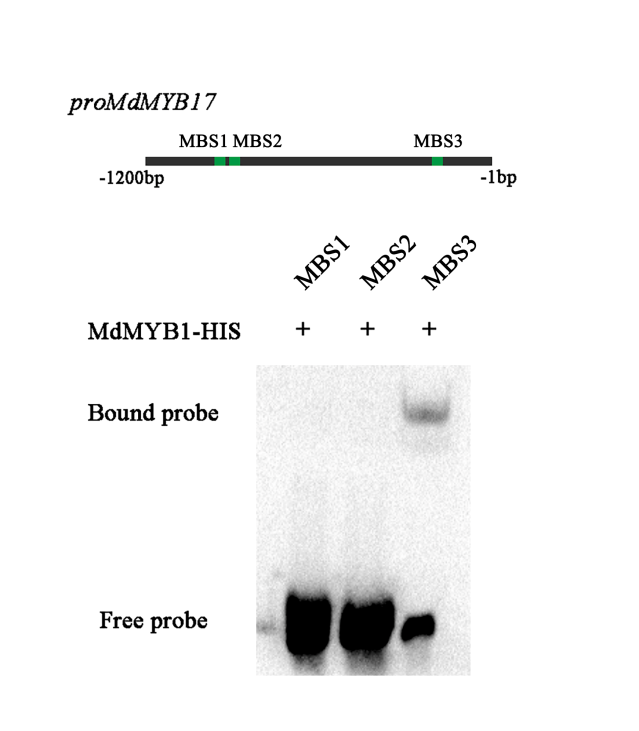


**Fig. S6:** Schematic diagram of the *MdMYB17* promoter. Green rectangles (MBS1, MBS2, and MBS3) indicate the potential MdMYB1-binding motif in the promoter. EMSA analysis indicating that MdMYB1 binds to the MBS3 motif in the *MdMYB17* promoter.

**
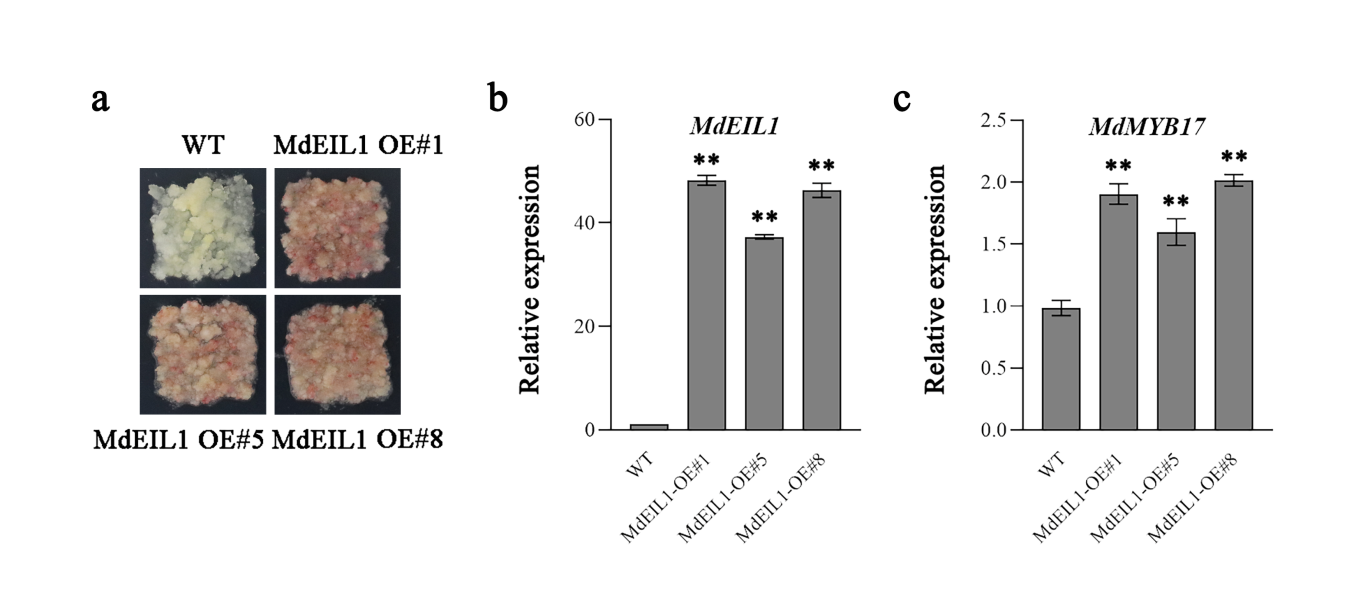
Fig. S7:** MdEIL1 activates anthocyanin synthesis and *MdMYB17* expression in ‘Orin’ apple calli. **a** Phenotypes of ‘Orin’ apple calli incubated at 14 °C and light（20,000 lux） for 10 days. WT: wild-type; *MdEIL1*-OE: *MdEIL1*-overexpression. **(b and c)** Expression levels of *MdEIL1* **(b)** and *MdMYB17* **(c)** in apple calli. Data are presented as the mean ± SD of three independent biological replicates. Asterisks indicate significant differences, as determined by Student’s *t*-test (**P* < 0.05, ***P* < 0.01).


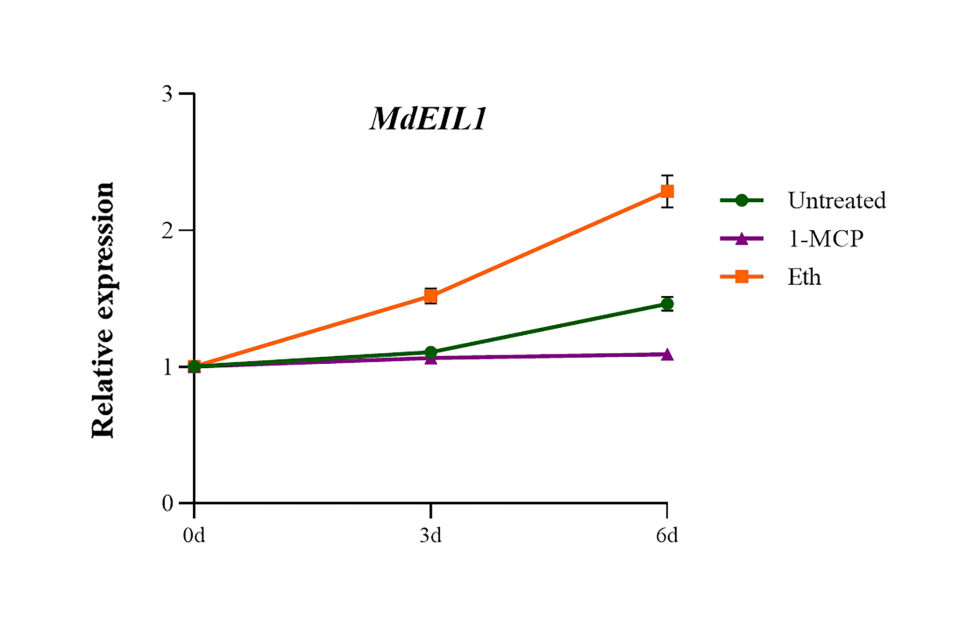


**Fig. S8:** Expression levels of *MdEIL1* in ‘Geneva Early’ apple fruit treated with ethephon and 1-MCP.


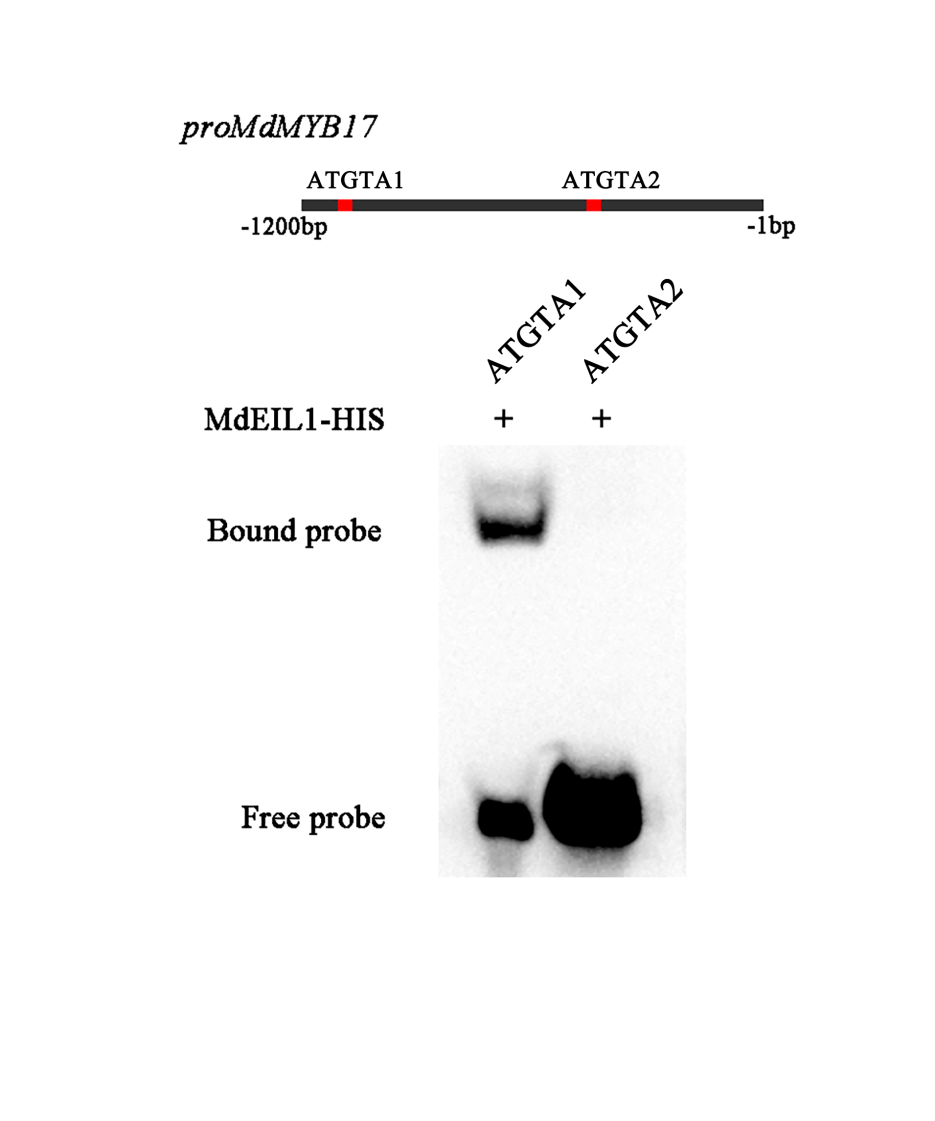


**Fig. S9:** Schematic diagram of the *MdMYB17* promoter. Red rectangles (ATGTA1 and ATGTA2) indicate the potential MdEIL1-binding motif. EMSA analysis indicating that MdEIL1 binds to the ATGTA1 motif in the *MdMYB17* promoter.


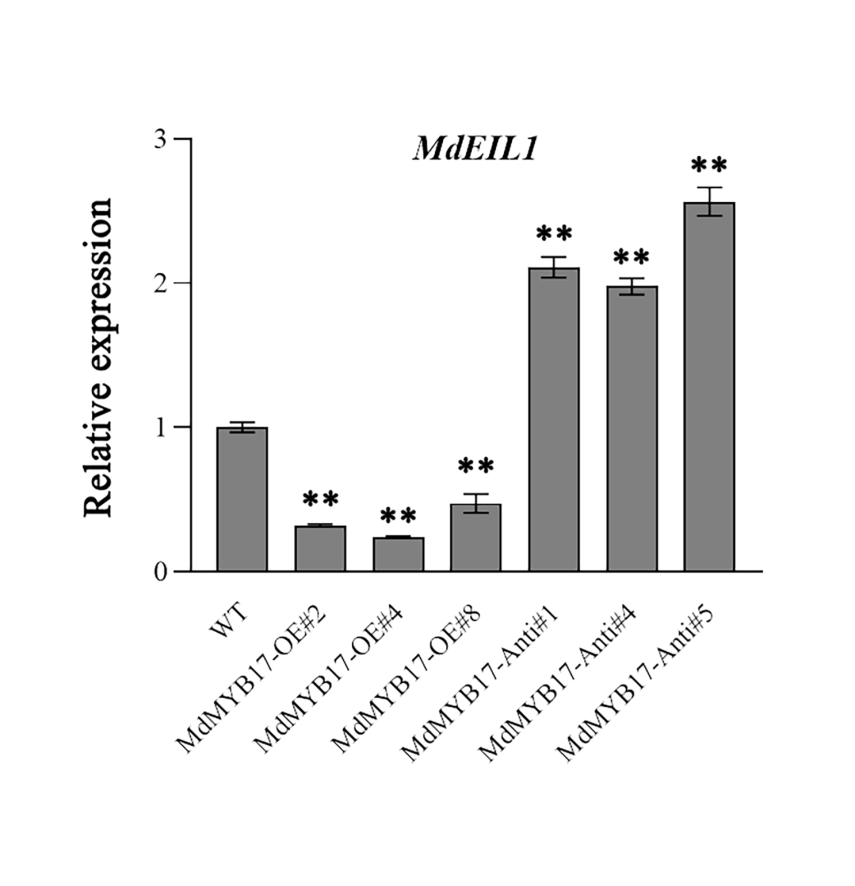


**Fig. S10:** *MdEIL1* expression level in ‘Orin’ apple calli. WT: wild-type; *MdMYB17*-OE: *MdMYB17*-overexpression; *MdMYB17*-Anti: *MdMYB17*-antisense suppression. Data are presented as the mean ± SD of three independent biological replicates. Asterisks indicate significant differences, as determined by Student’s *t*-test (**P* < 0.05, ***P* < 0.01).


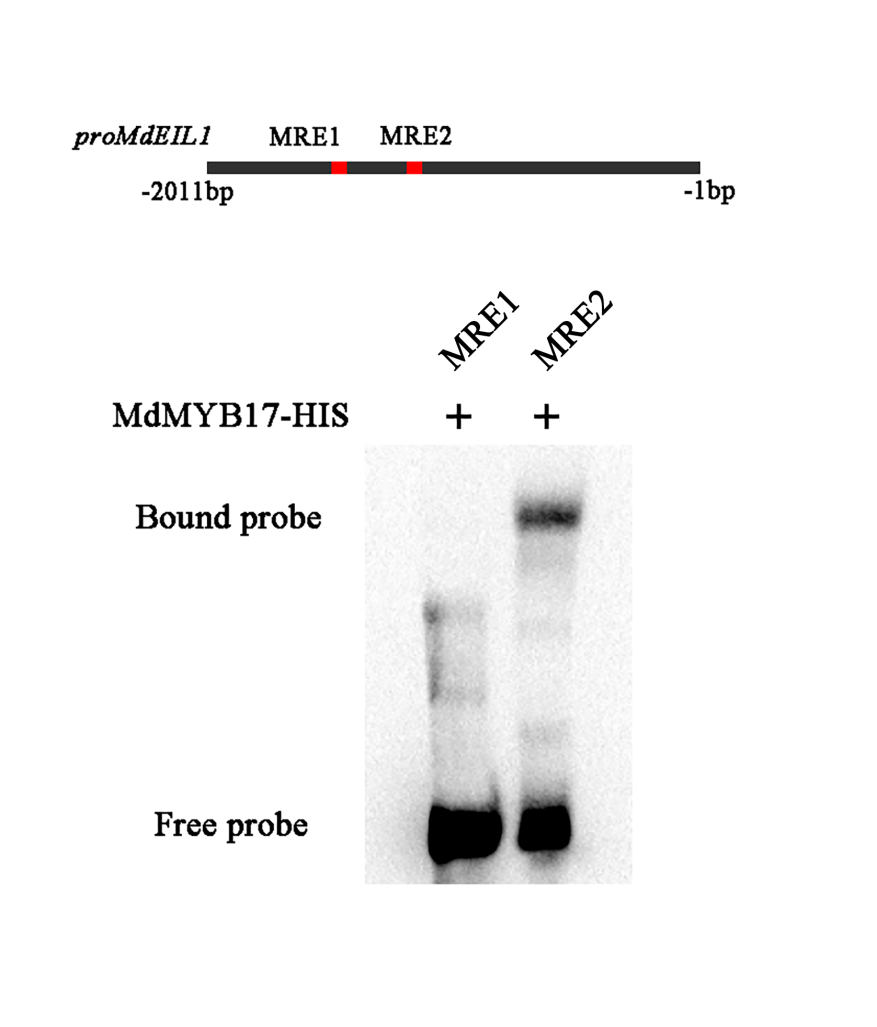


**Fig. S11:** Schematic diagram of the *MdEIL1* promoter. Red rectangles (MRE1 and MRE2) indicate the potential MdMYB17-binding motifs. EMSA analysis indicating that MdMYB17 binds to the MRE2 motif in the *MdEIL1* promoter.

| **Table S1 Primers used in this study.** | | |
| --- | --- | --- |
| **Primer Name** | **Sequence** | **Purpose** |
| MdActin-F | TGACCGAATGAGCAAGGAAATTACT | qRT-PCR |
| MdActin-R | TACTCAGCTTTGGCAATCCACATC |  |
| MdMYB17-F | CCAATAACCATCGCATAGG |  |
| MdMYB17-R | AAGATTAAGGTCAGGCAAAC |  |
| MdMYB16-F | CATAACCAATGGGCTTGTT |  |
| MdMYB16-R | ACTCTTCAAACCGTCACT |  |
| MdMYB308L-F | CCCTCTAAACCTGTTAATTCC |  |
| MdMYB308L-R | GCCAATCGTGAGATCAAG |  |
| MdMYB308-F | ACCCAGTGAGAGTTTGAA |  |
| MdMYB308-R | TGTAATCCAAGACCCCATT |  |
| MdMYB57-F | CACCACAACCGAAGAAGA |  |
| MdMYB57-R | TCTGCTGTAGTCTTGACTC |  |
| MdMYB109-F | CTGCGAGTGTTCTTGAAG |  |
| MdMYB109-R | AATGCTGGAGGTTTGTTTC |  |
| MdUFGT-F | CCACCGCCCTTCCAAACACTCT |  |
| MdUFGT-R | CACCCTTATGTTACGCGGCATGT |  |
| MdDFR-F | GATAGGGTTTGAGTTCAAGTA |  |
| MdDFR-R | TCTCCTCAGCAGCCTCAGTTTTCT |  |
| MdANS-F | CCAAGTGAAGCGGGTTGTGCT |  |
| MdANS-R | CAAAGCAGGCGGACAGGAGTAGC |  |
| MdMYB1-F | TGCCTGGACTCGAGAGGAAGACA |  |
| MdMYB1-R | CCTGTTTCCCAAAAGCCTGTGAA |  |
| MdMYB9-F | GCGATGATGACGACATTG |  |
| MdMYB9-R | CCTCAGAGTCAAGCAAGA |  |
| MdMYB11-F | AGGCGGTGAATAATCCAA |  |
| MdMYB11-R | GTTATAGAGCACAGAGAAATCG |  |
| MdEIL1-F | GTTTGATGCTTTGGGACTT |  |
| MdEIL1-R | CTGGTTCACTGGTTGTTG |  |
| MdMYB17-pRI101-F | GTCGACATGAGGAAACCTTGCTGTGAG | Primers for gene transgene |
| MdMYB17-pRI101-R | CCCGGGACTTGGAAGGCCTATTGTGAGA |  |
| MdMYB1-pRI101-F | GTCGACATGGAGGGATATAACGAAAACCT |  |
| MdMYB1-pRI101-R | CCCGGGTTCTTCTTTTGAATGATTCCAAAG |  |
| MdEIL1-pRI101-F | GTCGACATGGTGATCTTTGAGGAGTTAGGT |  |
| MdE1L1-pRI101-R | CCCGGGTGGGAACCAGGGGTCTT |  |
| MdMYB17-pFGC1008-F | AGGCGCGCCGGCAACTTTGGTGAAGATGAA |  |
| MdMYB17-pFGC1008-R | ATTTAAATACTTGGAAGGCCTATTGTGAGA |  |
| MdMYB17-pFGC1008-F | GGACTAGTGGCAACTTTGGTGAAGATGAA |  |
| MdMYB17-pFGC1008-R | CGGGATCCACTTGGAAGGCCTATTGTGAGA |  |
| MdMYB17-pHBT-F | GGATCCATGAGGAAACCTTGCTGTGAG | Luciferase reporter assay  Luciferase reporter assay |
| MdMYB17-pHBT-R | AGGCCTACTTGGAAGGCCTATTGTGAGA |  |
| MdMYB17m-pHBT-F | GGATCCATGAGGAAACCTTGCTGTGAG |  |
| MdMYB17m-pHBT-R | AGGCCTTGGTTTCTGATGATCATCAGAGTC |  |
| MdMYB1-pHBT-F | GGATCCATGGAGGGATATAACGAAAACCT |  |
| MdMYB1-pHBT-R | AGGCCTTTCTTCTTTTGAATGATTCCAAAG |  |
| MdE1L1-pHBT-F | GGATCCATGGTGATCTTTGAGGAGTTAGGT |  |
| MdE1L1-pHBT-R | AGGCCTTGGGAACCAGGGGTCTT |  |
| MdMYB1-pFRK-F | GGATCCTCATCTCTCTACTGCAGTGCCTAG |  |
| MdMYB1-pFRK-R | CCATGGCTCTTATCTGCTAGCAGCTAAGCTTA |  |
| MdDFR-pFRK-F | GGATCCAGTGAACAGTAACGAGAGTGTTTC |  |
| MdDFR-pFRK-R | CCATGGAGGTACTTGGAATAGTTGCTCCG |  |
| MdUFGT-pFRK-F | GGATCCCTGGGTTGAGAGAAGTCTCATGT |  |
| MdUFGT-pFRK-R | CCATGGTACAGCTTACAAGGCTAATCAGAAA |  |
| MdMYB17-pFRK-F | GGATCCCAAATATTTAAATTGGATTGAGATCC |  |
| MdMYB17-pFRK-R | CCATGGTACAGTAGTAATATTGAAGTTCTTCTTA |  |
| MdEIL1-pFRK-F | GGATCCATAAAAAAGATTTGACGGTTCAAA |  |
| MdEIL1-pFRK-R | CCATGGTTAGCACCGACACAAACCA |  |
| MdMYB17-pTRV2-F | GGATCCGGCAACTTTGGTGAAGATGAA | Transient infection |
| MdMYB17-pTRV2-R | CCCGGGACTTGGAAGGCCTATTGTGAGA |  |
| MdMYB17-pGADT7-F | CATATGATGAGGAAACCTTGCTGTGAG | Y1H assay |
| MdMYB17-pGADT7-R | GGATCCACTTGGAAGGCCTATTGTGAGA |  |
| MdMYB1-pGADT7-F | CATATGATGGAGGGATATAACGAAAACCT |  |
| MdMYB1-pGADT7-R | GGATCCTTCTTCTTTTGAATGATTCCAAAG |  |
| MdEIL1-pGADT7-F | CATATGATGGTGATCTTTGAGGAGTTAGGT |  |
| MdEIL1-pGADT7-R | GGATCCTGGGAACCAGGGGTCTT |  |
| MdMYB17-pHIS2-F | GAATTCCAAATATTTAAATTGGATTGAGATCC |  |
| MdMYB17-pHIS2-R | GAGCTCTACAGTAGTAATATTGAAGTTCTTCTTA |  |
| MdMYB1-pHIS2-F | GAATTCTCATCTCTCTACTGCAGTGCCTAG |  |
| MdMYB1-pHIS2-R | GAGCTCCTCTTATCTGCTAGCAGCTAAGCTTA |  |
| MdDFR-pHIS2-F | GAATTCAGTGAACAGTAACGAGAGTGTTTC |  |
| MdDFR-pHIS2-R | GAGCTCAGGTACTTGGAATAGTTGCTCCG |  |
| MdEIL1-pHIS2-F | GAATTCATAAAAAAGATTTGACGGTTCAAA |  |
| MdEIL1-pHIS2-R | GAGCTCTTAGCACCGACACAAACCA |  |
| MdUFGT-pHIS2-F | GAATTCCTGGGTTGAGAGAAGTCTCATGT |  |
| MdUFGT-pHIS2-R | GAGCTCTACAGCTTACAAGGCTAATCAGAAA |  |
| MdMYB17-pET32a-F | GGATTCATGAGGAAACCTTGCTGTGAG | EMSA assay  EMSA assay  EMSA assay |
| MdMYB17-pET32a-R | GTCGACACTTGGAAGGCCTATTGTGAGA |  |
| MdMYB1-pET32a-F | GGATTCATGGAGGGATATAACGAAAACCT |  |
| MdMYB1-pET32a-R | GTCGACTTCTTCTTTTGAATGATTCCAAAG |  |
| MdEIL1-pET32a-F | GGATTCATGGTGATCTTTGAGGAGTTAGGT |  |
| MdEIL1-pET32a-R | GTCGACTGGGAACCAGGGGTCTT |  |
| MdMYB1-MBS-Probe-F | AGACTGGTAGCTATTAACAACTGTTGGAATGTTTTAAACT |  |
| MdMYB1-MBS-Probe-R | AGTTTAAAACATTCCAACAGTTGTTAATAGCTACCAGTCT |  |
| MdMYB1-MBS-Mutated-F | AGACTGGTAGCTATTAACGACTATTGGAATGTTTTAAACT |  |
| MdMYB1-MBS-Mutated-R | AGTTTAAAACATTCCAATAGTCGTTAATAGCTACCAGTCT |  |
| MdDFR-MBS-Probe-F | AAACTCCCTAAATAAACCAGTTGTACTGGTGGATAGGCCT |  |
| MdDFR-MBS-Probe-R | AGGCCTATCCACCAGTACAACTGGTTTATTTAGGGAGTTT |  |
| MdDFR-MBS-Mutated-F | AAACTCCCTAAATAAACCGGTCGTACTGGTGGATAGGCCT |  |
| MdDFR-MBS-Mutated-R | AGGCCTATCCACCAGTACGACCGGTTTATTTAGGGAGTTT |  |
| MdUFGT-MBS-Probe-F | GTCAAGTATGGTACAAACAACTGCAATAAAATATTGCCAC |  |
| MdUFGT-MBS-Probe-R | GTGGCAATATTTTATTGCAGTTGTTTGTACCATACTTGAC |  |
| MdUFGT-MBS-Mutated-F | GTCAAGTATGGTACAAACGACTACAATAAAATATTGCCAC |  |
| MdUFGT-MBS-Mutated-R | GTGGCAATATTTTATTGTAGTCGTTTGTACCATACTTGAC |  |
| MdMYB17 ATGTA1-Probe-F | TCAAATTAATTATAAATTAAATGTAACCATGACAAGGAAAGTGTC |  |
| MdMYB17 ATGTA1-Probe-R | GACACTTTCCTTGTCATGGTTACATTTAATTTATAATTAATTTGA |  |
| MdMYB17 ATGTA-1-Mutated-F | TCAAATTAATTATAAATTAACTGTCACCATGACAAGGAAAGTGTC |  |
| MdMYB17 ATGTA1-Mutated-R | GACACTTTCCTTGTCATGGTGACAGTTAATTTATAATTAATTTGA |  |
| MdMYB17 ATGTA2-Probe-F | TTCCAAATTGTATGATTTTTTACATATATGATATTTAGATGATGA |  |
| MdMYB17 ATGTA2-Probe-R | TCATCATCTAAATATCATATATGTAAAAAATCATACAATTTGGAA |  |
| MdMYB17 ATGTA2-Mutated-F | TTCCAAATTGTATGATTTTTGACAGATATGATATTTAGATGATGA |  |
| MdMYB17 ATGTA2-Mutated-R | TCATCATCTAAATATCATATCTGTCAAAAATCATACAATTTGGAA |  |
| MdMYB17 MBS1-Probe-F | GTTAAATATCATGTGTACCACAGTTGCTTAGTAAAAAAAATGCCCT |  |
| MdMYB17 MBS1-Probe-R | AGGGCATTTTTTTTACTAAGCAACTGTGGTACACATGATATTTAAC |  |
| MdMYB17 MBS1-Mutated-F | GTTAAATATCATGTGTACCA TAGTCGCTTAGTAAAAAAAATGCCCT |  |
| MdMYB17 MBS1-Mutated-R | AGGGCATTTTTTTTACTAAGCGACTATGGTACACATGATATTTAAC |  |
| MdMYB17 MBS-2-Probe-F | ATGCCCTTGGTTTTATTTTTCAGTTGAAGAAACCAATTATCCGCAA |  |
| MdMYB17 MBS2-Probe-R | TTGCGGATAATTGGTTTCTTCAACTGAAAAATAAAACCAAGGGCAT |  |
| MdMYB17 MBS2-Mutated-F | ATGCCCTTGGTTTTATTTTTTAGTCGAAGAAACCAATTATCCGCAA |  |
| MdMYB17 MBS2-Mutated-R | TTGCGGATAATTGGTTTCTTCGACTAAAAAATAAAACCAAGGGCAT |  |
| MdMYB17 MBS3-Probe-F | AGATGTGTAGTTAAATTACCCAACTGTAAGTCAAGCGGAGTAGTCA |  |
| MdMYB17 MBS3-Probe-R | TGACTACTCCGCTTGACTTACAGTTGGGTAATTTAACTACACATCT |  |
| MdMYB17 MBS3-Mutated-F | AGATGTGTAGTTAAATTACCCGACTATAAGTCAAGCGGAGTAGTCA |  |
| MdMYB17 MBS3-Mutated-R | TGACTACTCCGCTTGACTTATAGTCGGGTAATTTAACTACACATCT |  |
| MdEIL1 MRE1-Probe-F | AACCGCGGTTAAAACGTAACCTAACCCTAGTTAAATAGGTC |  |
| MdEIL1 MRE1-Probe-R | GACCTATTTAACTAGGGTTAGGTTACGTTTTAACCGCGGTT |  |
| MdEIL1 MRE1-Mutated-F | AACCGCGGTTAAAACGTTACTTGACCCTAGTTAAATAGGTC |  |
| MdEIL1 MRE1-Mutated-R | GACCTATTTAACTAGGGTCAAGTAACGTTTTAACCGCGGTT |  |
| MdEIL1 MRE2-Probe-F | ATTGTTCTGTTTATTGTTTAGGTTAATCTGTTCTTATCTGC |  |
| MdEIL1 MRE2-Probe-R | GCAGATAAGAACAGATTAACCTAAACAATAAACAGAACAAT |  |
| MdEIL1 MRE2-Mutated-F | ATTGTTCTGTTTATTGTTCAAGTAAATCTGTTCTTATCTGC |  |
| MdEIL1 MRE2-Mutated-R | GCAGATAAGAACAGATTTACTTGAACAATAAACAGAACAAT |  |
| MdMYB17-pRI101-F | GTCGACATGAGGAAACCTTGCTGTGAG | ChIP-  qPCR assay |
| MdMYB17-pRI101-R | CCCGGGACTTGGAAGGCCTATTGTGAGA |  |
| MdMYB1-pRI101-F | GTCGACATGGAGGGATATAACGAAAACCT |  |
| MdMYB1-pRI101-R | CCCGGGTTCTTCTTTTGAATGATTCCAAAG |  |
| MdEIL1-pRI101-F | GTCGACATGGTGATCTTTGAGGAGTTAGGT |  |
| MdE1L1-pRI101-R | CCCGGGTGGGAACCAGGGGTCTT |  |
| proMdMYB1-F | TTTCCCTTGTCAATGTTGTC |  |
| proMdMYB1-R | ATATCCACAGAAGCAAACAC |  |
| proMdDFR-F | CCAATAACCAAGTCCATCG |  |
| proMdDFR-R | GTTCGTGGCATTCTGATT |  |
| proMdUFGT-F | ATTGACCTCCTGGTTGAA |  |
| proMdUFGT-R | GTACTGAGAGCTTTTCGTTA |  |
| proMdEIL1-MRE2-F | GGTACCTTCATTTTCTGCTT |  |
| proMdEIL1-MRE2-R | GAACCAAACAGATCAACCAT |  |
| proMdMYB17-MBS3-F | AGCATAGTACCAGACACC |  |
| proMdMYB17-MBS3-R | GGGGCTAGAGAACAATATAAC |  |
| proMdMYB17-ATGTA1-F | AGGAAATAAGAGAAACAATAGACC |  |
| proMdMYB17-ATGTA1-R | TCGAGTGCTAATAACACAATG |  |
| MdMYB17-pGBKT7-F | CATATGATGAGGAAACCTTGCTGTGAG | Y2H assay |
| MdMYB17-pGBKT7-R | GGATCCACTTGGAAGGCCTATTGTGAGA |  |
| MdEIL1-pGADT7-F | CATATGATGGTGATCTTTGAGGAGTTAGGT |  |
| MdEIL1-pGADT7-R | GGATCCTGGGAACCAGGGGTCTT |  |
| MdMYB17-NYFP-F | GGATCCATGAGGAAACCTTGCTGTGAG | BiFC assay |
| MdMYB17-NYFP-R | CTCGAGACTTGGAAGGCCTATTGTGAGA |  |
| MdEIL1-CYFP-F | GGATCCATGGTGATCTTTGAGGAGTTAGGT |  |
| MdEIL1-CYFP-R | CTCGAGTGGGAACCAGGGGTCTT |  |
| MdMYB17-pET32a-F | GGATTCATGAGGAAACCTTGCTGTGAG | Pull-down assay |
| MdMYB17-pET32a-R | GTCGACACTTGGAAGGCCTATTGTGAGA |  |
| MdEIL1-pGEX4T-1-F | GGATTCATGGTGATCTTTGAGGAGTTAGGT |  |
| MdEIL1-pGEX4T-1-R | GTCGACTGGGAACCAGGGGTCTT |  |
| MdMYB17-pHBT-F | GGATCCATGAGGAAACCTTGCTGTGAG | Co-IP assay |
| MdMYB17-pHBT-R | AGGCCTACTTGGAAGGCCTATTGTGAG |  |
| MdEIL1-pHBT-F | GGATCCATGGTGATCTTTGAGGAGTTAGGT |  |
| MdEIL1-pHBT-R | AGGCCTTGGGAACCAGGGGTCTT |  |

**Table S2 Genbank accession numbers of subgroup 4 R2R3-MYB TFs.**

| Name | Accession number |
| --- | --- |
| MdMYB16 | NP_001315806.1 |
| MdMYB17 | NP_001280904.1 |
| MdMYB109 | AHG99475.1 |
| MdMYB57 | XP_008378762 |
| MdMYB308 | XP_008377747 |
| MdMYB308L | XP_008374824 |
| AtMYB4 | AF062860.2 |
| AtMYB7 | NP_179263.1 |
| VvMYBC2-L1 | NM_001281204.1 |
| PtrMYB182 | AJI76863.1 |
